# PRMT1 Promotes the Self‐renewal of Leukemia Stem Cells by Regulating Protein Synthesis

**DOI:** 10.1002/advs.202308586

**Published:** 2024-12-12

**Authors:** Min Zhou, Yi Huang, Ping Xu, Shuyi Li, Chen Duan, Xiaoying Lin, Shilai Bao, Waiyi Zou, Jingxuan Pan, Chang Liu, Yanli Jin

**Affiliations:** ^1^ State Key Laboratory of Bioactive Molecules and Druggability Assessment Jinan University Guangzhou 510632 China; ^2^ Jinan University Institute of Tumor Pharmacology College of Pharmacy Jinan University Guangzhou 510632 China; ^3^ State Key Laboratory of Molecular Developmental Biology Institute of Genetics and Developmental Biology Chinese Academy of Sciences Beijing 100101 China; ^4^ School of Life Sciences University of Chinese Academy of Sciences Beijing 100049 China; ^5^ Department of Hematology The First Affiliated Hospital Sun Yat‐sen University Guangzhou 510080 China; ^6^ State Key Laboratory of Ophthalmology Zhongshan Ophthalmic Center Sun Yat‐sen University Guangzhou 510060 China

**Keywords:** CML, leukemia stem cells, PRMT1, protein synthesis, RPL29, self‐renewal

## Abstract

The application of tyrosine kinase inhibitors (TKIs) has revolutionized the management of chronic myeloid leukemia (CML). However, disease relapse and progression particularly due to persistent leukemia stem cells (LSCs) remain a big challenge in the clinic. Therefore, validation of the therapeutic vulnerability in LSCs is urgently needed. This study verifies the critical role of protein arginine methyltransferase 1 (PRMT1) in the maintenance of CML LSCs. It is found that PRMT1 promotes the survival and serially plating abilities of human primary CML LSCs. Genetic deletion of *Prmt1* significantly delays the leukemogenesis and impairs the self‐renewal of LSCs in BCR‐ABL**–**driven CML mice. PRMT1 regulates LSCs and leukemia development depending on its methyltransferase activity. Pharmacological inhibition of PRMT1 activity by MS023 remarkably eliminates LSCs and prolongs the survival of CML mice. Mechanistical studies reveal that PRMT1 promotes transcriptional activation of ribosomal protein L29 (RPL29) via catalyzing asymmetric dimethylation of histone H4R3 (H4R3me2a) at its gene promoter region. PRMT1 augments the global protein synthesis via RPL29 in CML LSCs. Taken together, the findings provide new evidence that histone arginine methylation modification regulates protein synthesis in LSCs and highlight PRMT1 as a valuable druggable target for patients with CML.

## Graphical abstract

This study indicates that PRMT1 deletion delays the initiation and propagation of leukemia as well as impairs the self‐renewal of CML LSCs. PRMT1 augments the global protein synthesis via H4R3me2a‐mediated transcriptional activation of RPL29 in CML LSCs, allowing for their maintenance. These findings identify PRMT1 as a promising therapeutic target in patients with CML.

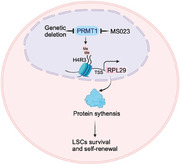

## 1 Introduction

Chronic myeloid leukemia (CML) is a common hematological cancer caused by the genetic translocation t (9;22) (q34; q11.2) generating the BCR‐ABL fusion oncogene. This fusion oncogene encodes BCR‐ABL oncoprotein with constitutive tyrosine kinase activity which can potently transform hematopoietic stem cells (HSCs) into leukemia stem cells (LSCs).^[^ ^1^ ^]^ The first‐line drugs, tyrosine kinase inhibitors (TKIs), such as imatinib, nilotinib, dasatinib, and olverembatinib, are highly effective in the treatment of most patients with CML, leading to a normal life expectancy.^[^ ^2^ ^]^ Nevertheless, disease relapse and progression possibly driven by the persistent LSCs remain a big challenge in the clinic.^[^ ^3^ ^]^

LSCs are considered a rare population of cells with the properties of self‐renewal capacity (stemness), differentiation disorder, and quiescent state.^[^ ^4^ ^]^ Emerging evidence demonstrates that the function of LSCs is independent of BCR‐ABL kinase activity. Hence, TKIs can effectively control but cannot cure CML because TKIs fail to eradicate LSCs.^[^ ^5^, ^6^ ^]^ The persistence of LSCs is responsible for the initiation, maintenance, and recurrence of CML disease. Therefore, the eradication of LSCs may be a hopeful strategy for radical treatment of CML.^[^ ^7^ ^]^ Currently, several pathways or regulators that uniquely maintain LSCs have been identified, including B‐cell lymphoma 2 (Bcl‐2) and myeloid cell leukemia 1 (Mcl‐1), Wnt/β‐catenin and Hedgehog pathways, arachidonate 5‐lipoxygenase (Alox5) and stearoyl– coenzyme A desaturase 1 (Scd1), p53 and c‐Myc network, and the hematopoietic microenvironment.^[^ ^8^, ^9^ ^]^ However, the precise regulation of LSCs self‐renewal remains incompletely characterized.

Epigenetic modifications (DNA methylation, histone modifications, and RNA methylation) have been regarded as critical drivers in the maintenance and self‐renewal of LSCs.^[^ ^10^ ^]^ For example, overexpressed histone deacetylase SIRT1 is essential for the self‐renewal of CML CD34^+^ cells and inhibition of SIRT1 eliminates LSCs by activation of p53.^[^ ^11^ ^]^ Enhancers of zeste homologue 2 (EZH2), a critical component of polycomb repressive complex 2 (PRC2), are required for the maintenance of CML LSCs and genetic deletion of *Ezh2* blocks leukemia development in a CML mouse model.^[^ ^12^, ^13^ ^]^ Our group is devoted to exploring epigenetic pathways in regulating LSCs, such as protein lysine methyltransferase G9A and protein arginine methyltransferases 5 and 7 (PRMT5 and PRMT7).^[^ ^14^, ^15^, ^16^ ^]^ PRMTs catalyze the methylation on arginine residues at the histone tail or other non‐histone proteins and are classified into type I PRMTs (e.g., PRMT1‐4, PRMT6, and PRMT8), type II PRMTs (e.g., PRMT5 and PRMT9), and type III PRMT (PRMT7).^[^ ^17^ ^]^ PRMTs play important roles in numerous biological processes, such as gene transcription, RNA splicing, DNA damage response, stem cell biology, and tumorigenesis.^[^ ^18^ ^]^ Our previous work demonstrated the critical roles of PRMT5 and PRMT7 for the maintenance and self‐renewal of CML LSCs through different mechanisms.^[^ ^15^ ^]^ Besides, PRMT4 (CARM1) is essential for the initiation of mixed lineage leukemia (MLL)‐AF9–driven acute myeloid leukemia (AML).^[^ ^19^ ^]^ Therefore, targeting PRMTs is a potential therapeutic strategy for eradicating LSCs.

PRMT1 is the predominant type I arginine methyltransferase and is responsible for 85% of arginine methylation in mammalian cells.^[^ ^20^ ^]^ Among the PRMTs family, PRMT1 predominantly catalyzes asymmetric dimethylation of histone H4R3 (H4R3me2a) which correlates with transcriptional activation.^[^ ^21^ ^]^ Dysregulation of PRMT1 has been observed in several cancer types. For instance, PRMT1 promotes breast cancer metastasis,^[^ ^22^ ^]^ epithelial‐mesenchymal‐transition in non‐small cell lung cancer,^[^ ^23^ ^]^ and multiple myeloma tumorigenesis.^[^ ^24^ ^]^ Besides, PRMT1 promotes the maintenance of AML harboring FMS‐like receptor tyrosine kinase‐3 internal tandem duplication (FLT3‐ITD) mutation via catalyzing methylation of FLT3‐ITD at arginine residues 972/973 (R972/973).^[^ ^25^ ^]^ Furthermore, PRMT1 maintains the properties of tumor‐initiating cells in esophageal squamous cell carcinoma.^[^ ^26^ ^]^ However, the critical role of PRMT1 in maintaining CML LSCs remains elusive.

In this study, we determined the role of PRMT1 in the maintenance and self‐renewal of CML LSCs. The highly expressed PRMT1 enhanced the survival and serially plating capacities of human primary CML LSCs. Genetic deletion of *Prmt*1 blocked leukemia development and impaired the self‐renewal of LSCs in CML mice. Pharmacological inhibition of PRMT1 activity selectively eradicated LSCs. Mechanistically, we identified ribosomal protein L29 (RPL29) as a key downstream functional mediator of PRMT1. PRMT1 augmented global protein synthesis via transcriptional activation of RPL29 expression in CML LSCs. Our findings link histone arginine methylation with global protein synthesis in LSCs and identify PRMT1 as a promising target for the elimination of LSCs in CML.

## 2 Results

### 2.1 Elevated PRMT1 Promotes the Survival and Serially Plating Capacities of Human Primary CML LSCs

**Figure 1:**
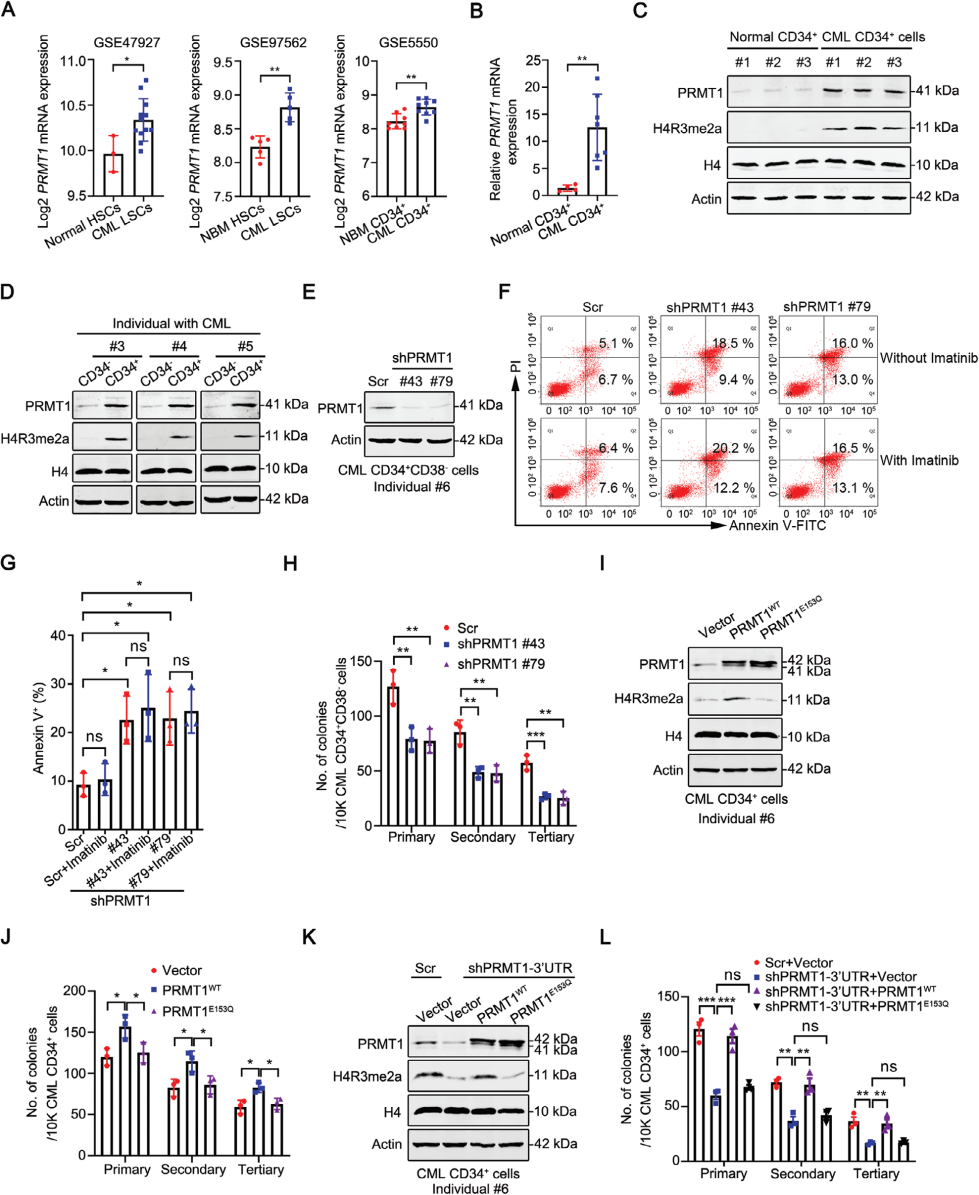
PRMT1 promotes the survival and serially plating abilities of human primary CML LSCs. A–D) *PRMT1* was highly expressed in CML LSCs. The mRNA expression of *PRMT1* in CML LSCs (CD34^+^CD38^−^) and normal HSCs (CD34^+^CD38^−^). Data are derived from GSE47927 (*left*) and GSE97562 (*middle*) datasets, respectively. The mRNA expression of *PRMT1* in CML CD34^+^ cells and normal CD34^+^ cells. Data are derived from GSE5550 (*right*) dataset (A). qRT‐PCR analysis of the mRNA expression of *PRMT1* in primary CD34^+^ cells from individuals with CML (*n =* 7) and normal CD34^+^ cells from healthy donors (*n =* 4) (B). Western blotting analysis of the protein levels of PRMT1 and H4R3me2a in CML CD34^+^ cells (*n =* 3) and normal CD34^+^ cells (*n =* 3) (C). Western blotting analysis of the protein levels of PRMT1 and H4R3me2a in CD34^+^ cells and CD34^−^ cells from the same individual with CML (*n =* 3) (D). E–G) Knockdown of *PRMT1* induced apoptosis in CML LSCs. CML CD34^+^CD38^−^ cells (*n =* 3) were transduced with Scramble (Scr), sh*PRMT1* #43, or sh*PRMT1* #79 lentivirus for 48 h, and then treated ± imatinib (2.5 µM) for 24 h. The apoptosis of CML CD34^+^CD38^−^ cells was detected by flow cytometry after staining with Annexin V‐FITC and PI. PRMT1 knockdown was confirmed by Western blotting assay (E). Representative flow cytometry histograms (F) and quantitative results for apoptotic cells (Annexin V^+^) (G) were shown. H) Knockdown of *PRMT1* inhibited the serially plating capacity of CML LSCs. The viable CML CD34^+^CD38^−^ cells (*n =* 3) with *PRMT1* knockdown were seeded in methylcellulose medium (MethoCult H4434) for 3 rounds of CFC/replating assay. I,J) PRMT1 regulated the serially plating ability of CML CD34^+^ cells depending on its methyltransferase activity. CML CD34^+^ cells (*n =* 3) were transduced with vector, wild‐type *PRMT1* (*PRMT1^WT^*) or enzymatic mutant *PRMT1* (*PRMT1^E153Q^*) lentivirus, and then 3 rounds of CFC/replating assay was performed. PRMT1 overexpression was examined by Western blotting analysis I). Quantitative results for CFC/replating assay were shown (J). K, L) CML CD34^+^ cells (*n =* 3) were transduced with shRNA lentivirus targeting *PRMT1* 3′UTR (sh*PRMT1*‐3′UTR) together with *PRMT1^WT^* or *PRMT1^E153Q^* lentivirus, and then 3 rounds of CFC/replating assay was performed. The expression of PRMT1 was examined by Western blotting analysis K). The quantitative results of CFC/replating assay were shown (L). Data are represented as means ± SEM. ^*^ *p* < 0.05, ^**^ *p* < 0.01, ^***^ *p* < 0.001; ns, not significant by Student's *t* test (A and B) or one‐way ANOVA with Tukey's test (G, H, J and L).

To investigate the role of PRMT1 in CML LSCs (CD34^+^CD38^−^),^[^ ^27^ ^]^ we first analyzed the expression of PRMT1 in CML LSCs and normal HSCs (CD34^+^CD38^−^)^[^ ^27^ ^]^ derived from publicly available databases. The results showed that *PRMT1* was highly expressed in CML LSCs compared with normal HSCs (**Figure** **1**A). Consistently, the mRNA expression of *PRMT1* was higher in leukemia stem/progenitor cells (LSPCs) sorted from individuals with CML (CML CD34^+^) than in hematopoietic stem/progenitor cells (HSPCs) sorted from healthy donors (normal CD34^+^) (Figure 1B). Furthermore, the protein levels of PRMT1 and H4R3me2a, its histone methylation marker, were dramatically increased in CML CD34^+^ cells compared with normal CD34^+^ cells as detected by Western blotting analysis (Figure 1C). Besides, we also examined the expression of PRMT1 and H4R3me2a in CD34^+^ cells and CD34^−^ cells sorted from the same individual with CML. Our results showed that the protein levels of PRMT1 and H4R3me2a were specifically elevated in CML CD34^+^ cells but not CML CD34^−^ cells (Figure 1D). Overall, these data indicate that PRMT1 is overexpressed in CML LSCs.

To further determine the function of PRMT1 in CML LSCs, we silenced PRMT1 expression in primary CML CD34^+^CD38^−^ cells by using two independent short hairpin RNAs (shRNAs) (Figure 1E). Flow cytometry analysis showed that *PRMT1* knockdown significantly induced apoptosis in CML CD34^+^CD38^−^ cells, without an additive or synergistic effect when co‐treated with imatinib (Figure 1F,G). This may be due to the compensatory effects of other PRMTs (e.g., PRMT5, PRMT7) or unknown factors to impact the survival of CML LSCs. Moreover, the knockdown of *PRMT1* inhibited the colony‐forming cell (CFC) formation and replating ability of CML LSCs (Figure 1H). Conversely, overexpression of wild‐type (WT) *PRMT1* (*PRMT1^WT^*) rather than enzymatic mutant *PRMT1* (*PRMT1^E153Q^*)^[^ ^28^ ^]^ increased the serially plating ability of CML CD34^+^ cells (Figure 1I,J). In addition, we found that restoration of *PRMT1^WT^* but not *PRMT1^E153Q^* rescued the impaired CML progenitor activity mediated by *PRMT1* knockdown (Figure 1K,L), suggesting that PRMT1 enhances the serially plating capacity of CML CD34^+^ cells depending on its methyltransferase activity.

Accordingly, we selected a type I PRMTs inhibitor MS023 to test whether pharmacological inhibition of PRMT1 suppresses the function of LSCs.^[^ ^29^ ^]^ CML CD34^+^CD38^−^ cells were treated with MS023 ± imatinib for 48 h, and the apoptotic cells were measured by flow cytometry. As expected, MS023 treatment dramatically decreased the H4R3me2a level and imatinib reduced the p‐BCR‐ABL level in primary CML CD34^+^CD38^−^ cells (Figure S1A, Supporting Information). Similarly, MS023 exposure induced apoptosis in CML CD34^+^CD38^−^ cells, without an additive or synergistic effect when co‐treated with imatinib (Figure S1B,C, Supporting Information). MS023 treatment also suppressed the serially plating ability of CML CD34^+^CD38^−^ cells (Figure S1D, Supporting Information).

Collectively, these data demonstrate that PRMT1 plays a critical role in promoting the survival and serially plating abilities of human primary CML LSCs.

### 2.2 PRMT1 is Crucial for the leukemogenesis of BCR‐ABL–Driven CML Mice

**Figure 2:**
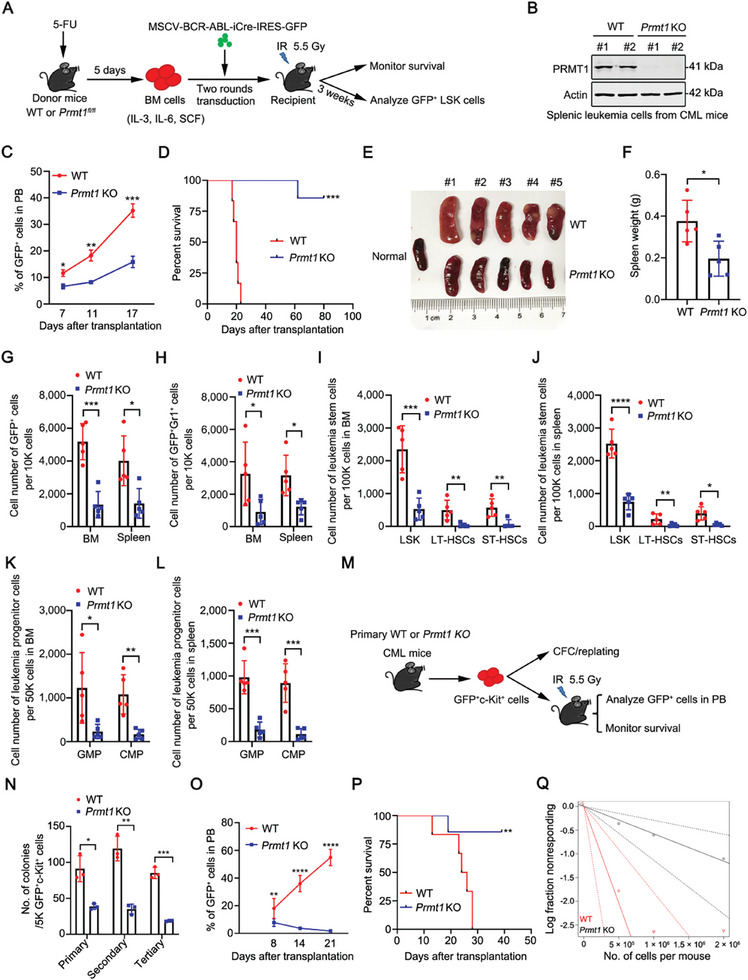
*Prmt1* knockout blocks leukemogenesis and impairs self‐renewal of LSCs in CML mice. A) Experimental scheme to obtain the WT or *Prmt1* knockout (KO) CML mice and detect the in vivo effect of *Prmt1* KO on LSCs. B) Western blotting analysis of the protein level of PRMT1 in splenic leukemia cells from WT or *Prmt1* KO CML mice. C–F) *Prmt1* KO delayed the development of CML. Flow cytometry analysis of the percentage of GFP^+^ cells in PB of recipient mice. *n =* 5 mice per group (C). Kaplan–Meier survival curves for recipients received BCR‐ABL‐iCre‐transduced BM cells from WT or *Prmt1* KO donor mice were shown. WT (*n =* 6), *Prmt1* KO (*n =* 7) (D). Representative photographs and the weight of spleens were shown. *n =* 5 mice for WT or *Prmt1* KO group (E, F). G–L) *Prmt1* KO decreased the leukemia burden and LSPCs in CML mice. The cell number of leukemia (GFP^+^) cells and leukemia myeloid (GFP^+^Gr1^+^) cells in 10K BM (G) or spleen (H) cells; The cell number of GFP^+^LSK cells (GFP^+^Lin^−^Sca‐1^+^c‐Kit^+^), GFP^+^ long‐term HSCs (GFP^+^LT‐HSCs, GFP^+^LSK Flt3^−^CD150^+^CD48^−^), GFP^+^ short‐term HSCs (GFP^+^ST‐HSCs, GFP^+^LSK Flt3^−^CD150^−^CD48^−^) in 100K BM (I) or spleen (J) cells; The cell number of GFP^+^ granulocyte‐macrophage progenitor (GFP^+^GMP, GFP^+^Lin^−^Sca‐1^−^c‐Kit^+^CD34^+^FcγRII/III^high^) and GFP^+^ common myeloid progenitor (GFP^+^CMP, GFP^+^Lin^−^Sca‐1^−^c‐Kit^+^CD34^+^FcγRII/III^low^) cells in 50K BM (K) or spleen (L) cells. *n =* 5 mice per group. M‐P) *Prmt1* KO attenuated the self‐renewal of CML LSCs. Experimental strategy to examine the impact of *Prmt1* KO on the self‐renewal of LSCs (M). GFP^+^c‐Kit^+^ cells (5000 cells/well) sorted from primary WT or *Prmt1* KO CML mice (*n =* 3) were seeded in MethoCult M3434 methylcellulose medium for 3 rounds of CFC/replating assay N). WT or *Prmt1* KO GFP^+^c‐Kit^+^ cells (2 × 10^5^ cells/mouse) were transplanted into secondary sublethally irradiated (550 cGy) C57BL/6 mice. The percentages of GFP^+^ cells in PB on day 8, 14, and 21 were detected by flow cytometry O). Kaplan–Meier survival curves of secondary recipients were shown P). WT (*n =* 6), *Prmt1* KO (*n =* 7). Q) The frequency of LSCs was examined by in vivo limiting dilution assay. Data are represented as means ± SEM. ^*^ *p* < 0.05, ^**^ *p* < 0.01, ^***^ *p* < 0.001, ^****^ *p* < 0.0001; by Student's *t* test (C, F–L, N and O) or log‐rank test (D and P).

To validate the role of PRMT1 in leukemogenesis of BCR‐ABL**–**driven murine CML, we generated the floxed *Prmt1* mice (*Prmt1^fl/fl^*) (Figure S2A, Supporting Information). Next, we transduced the bone marrow (BM) cells from 5‐fluorouracil (5‐FU)‐treated WT or *Prmt1^fl/fl^* mice with BCR‐ABL‐iCre‐GFP retrovirus and transplanted them into sublethally irradiated recipients to induce CML after verifying the equal transduction efficiency of BCR‐ABL‐iCre‐GFP retrovirus (**Figure** **2**A; Figure S2B, Supporting Information). Recipients which received BCR‐ABL‐iCre‐GFP retrovirus‐transduced BM cells from *Prmt1^fl/fl^* or WT donor mice were referred to as *Prmt1* knockout (KO) or WT CML mice, respectively. The protein level of PRMT1 was obviously decreased in splenic leukemia cells from *Prmt1* KO CML mice relative to WT CML mice (Figure 2B).

Our results revealed that *Prmt1* KO blocked CML development, as demonstrated by the decreased percentage of GFP^+^ cells in peripheral blood (PB) on days 7, 11, and 17 post‐transplantation (Figure 2C). Consistently, *Prmt1* KO prolonged survival (median survival: WT vs *Prmt1* KO was 20 days vs not reached after monitoring for 80 days; Figure 2D), and ameliorated splenomegaly and spleen weight of CML mice (Figure 2E,F). Flow cytometry analysis showed that *Prmt1* KO significantly decreased the leukemia burden (cell number of GFP^+^ leukemia cells and GFP^+^Gr1^+^ myeloid cells) (Figure 2G,H; Figure S2C, Supporting Information), and the cell number of LSPCs including GFP^+^LSK cells, GFP^+^ long‐term HSCs (GFP^+^LT‐HSCs), GFP^+^ short‐term HSCs (GFP^+^ST‐HSCs) (Figure 2I,J; Figure S2D, Supporting Information), as well as GFP^+^ granulocyte‐macrophage progenitor (GFP^+^GMP) and GFP^+^ common myeloid progenitor (GFP^+^CMP) cells in CML mice (Figure 2K,L; Figure S2E, Supporting Information).

To further explore the role of PRMT1 in leukemogenesis, we generated tamoxifen‐inducible *Prmt1* KO mice (*Prmt1^fl/fl^*; *Cre‐ER^T2^*) by crossing *Prmt1^fl/fl^* mice with tamoxifen‐inducible Cre transgenic mice (Rosa26‐*Cre‐ER^T2^*). BM cells from tamoxifen‐treated *Prmt1^fl/fl^*; *Cre‐ER^T2^* or *Prmt1^fl/fl^* mice were transduced with BCR‐ABL‐GFP retrovirus and transplanted into sublethally irradiated recipients to induce CML (Figure S3A, Supporting Information). Tamoxifen treatment induced PRMT1 deletion in BM cells from *Prmt1^fl/fl^*; *Cre‐ER^T2^* mice compared with those from *Prmt1^fl/fl^* mice (Figure S3B, Supporting Information). Similarly, tamoxifen‐induced *Prmt1* deletion significantly prolonged the survival (median survival: *Prmt1^fl/fl^* vs *Prmt1^fl/fl^*; *Cre‐ER^T2^* was 20 days vs not reached after monitoring for 60 days; Figure S3C, Supporting Information), obviously alleviated the splenomegaly and spleen weight (Figure S3D,E, Supporting Information), reduced the leukemia burden and percentages of LSPCs in BM and spleen of CML mice (Figure S3F–M, Supporting Information).

To investigate whether PRMT1 maintains the self‐renewal of LSCs, we sorted GFP^+^c‐Kit^+^ cells from the primary WT or *Prmt1* KO CML mice and performed in vitro CFC/replating assay or in vivo disease reconstitution assay (Figure 2M). Our data showed that *Prmt1* KO inhibited the serially plating ability of GFP^+^c‐Kit^+^ cells (Figure 2N). Besides, the recipients which received *Prmt1* KO GFP^+^c‐Kit^+^ cells exhibited reduced leukemia burden (GFP^+^ cells) and prolonged survival (median survival: WT vs *Prmt1* KO was 24 days vs not reached after monitoring for 39 days) compared with those which received WT GFP^+^c‐Kit^+^ cells (Figure 2O,P). Moreover, the in vivo limiting dilution assay revealed that *Prmt1* KO markedly decreased the frequency of LSCs (Figure 2Q; Table S1, Supporting Information).

Taken together, these results suggest that *Prmt1* deletion delays leukemogenesis, eliminates LSCs, and impairs the self‐renewal of LSCs.

### 2.3 PRMT1 is Necessary for the Maintenance of BCR‐ABL–Driven CML Mice

To assess the effects of *Prmt1* loss on the propagation of leukemia, we transduced BM cells from 5‐FU‐treated *Prmt1^fl/fl^*; *Cre‐ER^T2^* or *Prmt1^fl/fl^* mice with BCR‐ABL‐GFP retrovirus and transplanted them into sublethally irradiated recipients. The recipients were administered tamoxifen (100 mg kg^−1^) for 4 times on days 7, 9, 11, and 13 post‐transplantation (Figure S4A, Supporting Information). Tamoxifen treatment effectively reduced PRMT1 expression in leukemia cells from *Prmt1^fl/fl^*; *Cre‐ER^T2^* CML mice (Figure S4B, Supporting Information). The results showed that tamoxifen‐induced *Prmt1* loss blocked leukemia development (Figure S4C, Supporting Information) and prolonged the survival of CML mice (median survival: *Prmt1^fl/fl^* vs *Prmt1^fl/fl^*; *Cre‐ER^T2^* was 14.5 days vs not reached after monitoring for 60 days; Figure S4D, Supporting Information).

To further determine the effects of *Prmt1* loss on the self‐renewal of LSCs, we sorted GFP^+^c‐Kit^+^ cells from the primary *Prmt1^fl/fl^*; *Cre‐ER^T2^* or *Prmt1^fl/fl^* CML mice and performed in vitro CFC/replating assay or in vivo disease reconstitution assay (Figure S4E, Supporting Information). Our data showed that *Prmt1* deletion inhibited the serially plating ability of GFP^+^c‐Kit^+^ cells (Figure S4F, Supporting Information). Besides, in vivo disease reconstitution assay indicated that the secondary *Prmt1‐*deleted CML mice had much lower leukemia burden (GFP^+^ cells; Figure S4G, Supporting Information) and prolonged survival (median survival: *Prmt1^fl/fl^* vs *Prmt1^fl/fl^; Cre‐ER^T2^* was 21 days vs not reached after monitoring for 65 days; Figure S4H, Supporting Information).

Taken together, these results indicate that PRMT1 is required for the propagation of leukemia and self‐renewal of LSCs.

### 2.4 PRMT1 is Critical for Leukemia Development in T315I BCR‐ABL–Driven CML Mice

T315I mutation in BCR‐ABL is a representative and stubborn point mutation that confers resistance to imatinib.^[^ ^30^ ^]^ To explore whether PRMT1 is also required for CML LSCs harboring T315I BCR‐ABL, we transduced BM cells from *Prmt1^fl/fl^*; *Cre‐ER^T2^* or *Prmt1^fl/fl^* mice with T315I‐BCR‐ABL‐GFP retrovirus and transplanted them into sublethally irradiated recipient mice to induce CML (Figure S5A, Supporting Information). The recipient mice which received T315I‐BCR‐ABL^+^ *Prmt1^fl/fl^*; *Cre‐ER^T2^* BM cells survived longer (median survival: *Prmt1^fl/fl^* vs *Prmt1^fl/fl^*; *Cre‐ER^T2^* was 22 vs 75 days; Figure S5B, Supporting Information), showed less severe splenomegaly and spleen weight (Figure S5C,D, Supporting Information) than those which received T315I‐BCR‐ABL^+^ *Prmt1^fl/fl^* BM cells. Consistently, the proportions of leukemia cells (T315I‐BCR‐ABL‐GFP^+^), myeloid cells (T315I‐BCR‐ABL‐GFP^+^Gr1^+^), LSPCs including T315I‐BCR‐ABL‐GFP^+^LSK cells, T315I‐BCR‐ABL‐GFP^+^LT‐HSCs and T315I‐BCR‐ABL‐GFP^+^ST‐HSCs, as well as T315I‐BCR‐ABL‐GFP^+^GMP and T315I BCR‐ABL‐GFP^+^CMP cells was significantly lower in BM and spleen of *Prmt1^fl/fl^*; *Cre‐ER^T2^* CML mice than *Prmt1^fl/fl^* CML mice (Figure S5E–K, Supporting Information). These results indicate that PRMT1 is essential for CML development induced by BCR‐ABL T315I mutation.

### 2.5 PRMT1 Regulates CML Development and LSCs Depending on its Methyltransferase Activity

To investigate whether PRMT1 regulates LSCs in a methyltransferase activity‐dependent manner, BM and spleen cells from primary WT CML mice transduced with pCDH‐vector, pCDH‐wild‐type *PRMT1* (*PRMT1^WT^*) or enzymatic mutant *PRMT1* (*PRMT1^E153Q^*) lentivirus were transplanted into secondary recipients to induce CML (Figure S6A, Supporting Information). PRMT1 overexpression in splenic leukemia cells from secondary CML mice was confirmed by Western blotting analysis (Figure S6B, Supporting Information). The recipient mice which received leukemia cells overexpressed with *PRMT1^WT^* but not *PRMT1^E153Q^* exhibited shorter survival compared with those which received leukemia cells overexpressed with empty vector (median survival: Vector vs *PRMT1^WT^* vs *PRMT1^E153Q^* was 25 vs 16 vs 25 days; Figure S6C, Supporting Information). Correspondingly, the recipient mice which received leukemia cells overexpressed with *PRMT1^E153Q^* showed less splenomegaly and spleen weight than those which received leukemia cells overexpressed with *PRMT1^WT^* (Figure S6D, E, Supporting Information). Overexpression of *PRMT1^WT^* but not *PRMT1^E153Q^* increased the leukemia burden and the percentages of LSPCs in BM and spleen of secondary CML mice (Figure S6F–L, Supporting Information).

**Figure 3:**
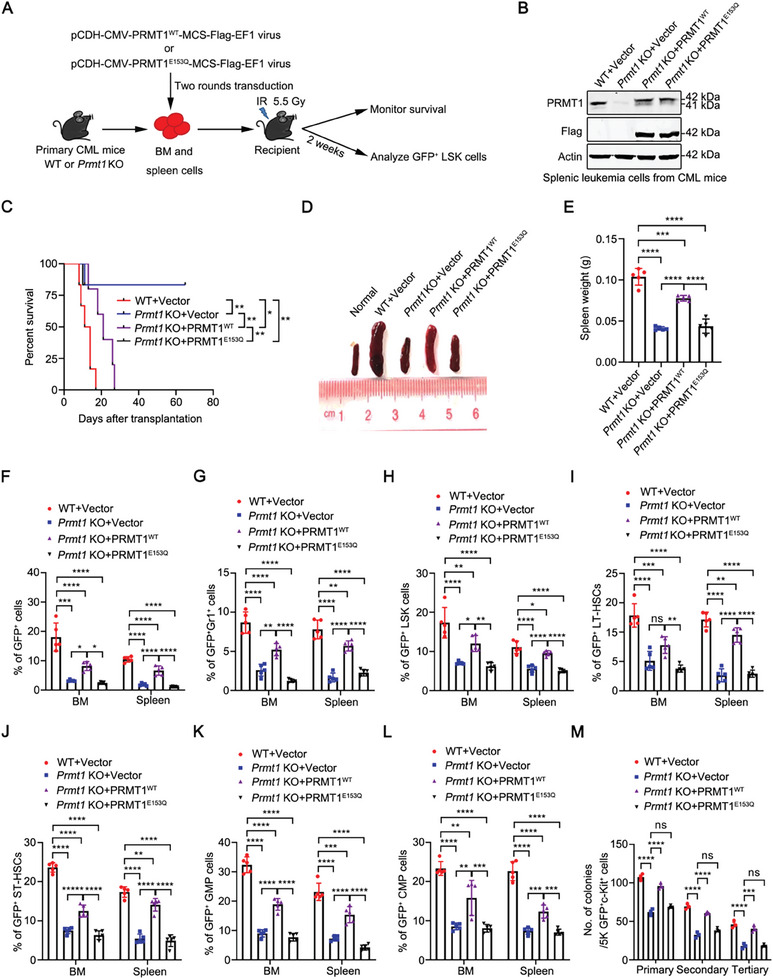
PRMT1 regulates LSCs depending on its methyltransferase activity. A) Experimental strategy to determine that PRMT1 regulates LSCs in a methyltransferase activity‐dependent manner. BM and spleen cells from the primary WT or *Prmt1* KO CML mice were transduced with lentivirus (Vector, *PRMT1^WT^* or *PRMT1^E153Q^*) and transplanted into the secondary recipients to induce CML. B) Western blotting analysis of the protein level of PRMT1 in splenic leukemia cells from secondary CML mice. C–L) Restoration of *PRMT1^WT^* rather than *PRMT1^E153Q^* rescued the *Prmt1* loss‐mediated alleviation of malignant phenotypes in CML mice. Kaplan–Meier survival curves of secondary recipients were shown (C). *n =* 5–6 mice per group. Representative photograph and the weight of spleens were shown (D, E). *n =* 5 mice per group. Flow cytometry analysis of the percentages of GFP^+^ cells (F), GFP^+^Gr1^+^ cells (G), GFP^+^LSK cells (H), GFP^+^LT‐HSCs (I), GFP^+^ST‐HSCs (J), GFP^+^GMP (K) and GFP^+^CMP cells (L) in BM and spleen were shown. *n =* 5 mice per group. M) PRMT1 regulates CML progenitor activity depending on its methyltransferase activity. The GFP^+^c‐Kit^+^ cells sorted from the secondary recipients were subjected to CFC/replating assay. Data are represented as means ± SEM. ^*^ *p* < 0.05, ^**^ *p* < 0.01, ^***^ *p* < 0.001, ^****^ *p* < 0.0001, ns, not significant, by log‐rank test (C) or one‐way ANOVA with Tukey's test (E–M).

To strengthen these findings, we transduced BM and spleen cells from primary WT or *Prmt1* KO CML mice with pCDH‐vector, pCDH‐*PRMT1^WT^* or pCDH‐*PRMT1^E153Q^* lentivirus and transplanted them into the secondary recipients to induce CML (**Figure** **3**A). Western blotting analysis showed that the WT or mutant PRMT1 was successfully restored (Figure 3B). The recipient mice that received leukemia cells reconstituted with *PRMT1^WT^* but not *PRMT1^E153Q^* died earlier than those that received leukemia cells reconstituted with empty vector (median survival: *Prmt1* KO+*PRMT1^WT^* vs *Prmt1* KO*+PRMT1^E153Q^* was 21 days vs not reached after monitoring for 65 days; Figure 3C). Correspondingly, the recipient mice which received leukemia cells reconstituted with *PRMT1^E153Q^* showed less splenomegaly and spleen weight than those which received leukemia cells reconstituted with *PRMT1^WT^* (Figure 3D,E). Restoration of *PRMT1^WT^* but not *PRMT1^E153Q^* reversed *Prmt1* KO‐mediated reductions of the leukemia burden and the percentages of LSPCs in BM and spleen of secondary CML mice (Figure 3F–L). In addition, we found that re‐introduction with WT *PRMT1* but not the mutant *PRMT1* reversed the *Prmt1* deletion‐mediated reduction of the serially plating ability of LSCs (Figure 3M). These results suggest that *PRMT1^WT^* rather than *PRMT1^E153Q^* rescues the impaired CML progenitor engraftment activity mediated by *Prmt1* loss.

### 2.6 Pharmacological Inhibition of PRMT1 Effectively Eliminates LSCs and Impairs the Self‐Renewal of LSCs in CML Mice

Next, we evaluated the effect of pharmacological inhibition of PRMT1 by MS023 on LSCs elimination in CML mice (Figure S7A, Supporting Information). The results demonstrated that MS023 treatment prolonged the survival (median survival: Vehicle vs MS023 was 14.5 days vs not reached after monitoring for 100 days; Figure S7B, Supporting Information), and alleviated the splenomegaly and spleen weight (Figure S7C,D, Supporting Information). Consistently, MS023 significantly decreased the leukemia burden and the populations of LSPCs in BM and spleen of CML mice relative to vehicle treatment (Figure S7E–J, Supporting Information). However, the proportions of GFP^−^LSK cells, GFP^−^LT‐HSCs, GFP^−^ST‐HSCs, GFP^−^GMP, and GFP^−^CMP cells in BM and spleen of CML mice treated with MS023 versus vehicle were not significantly altered (Figure S7K,L, Supporting Information). Besides, we examined the effect of *Prmt1* deletion on quiescent LSK cells by flow cytometry and found that *Prmt1* loss reduced the percentage of G_0_‐phase cells as defined by Ki67^low^ Hoechst3342^low^ population in LSK cells (Figure S7M, Supporting Information). Taken together, these results suggest that pharmacological inhibition of PRMT1 effectively eradicates LSCs, whereas does not damage normal hematopoiesis in CML mice.

To test the effect of pharmacological inhibition of PRMT1 on the self‐renewal of LSCs, we sorted GFP^+^c‐Kit^+^ cells from the vehicle or MS023 treated primary CML mice and performed in vitro CFC/replating assay or in vivo disease reconstitution assay (Figure S7N, Supporting Information). MS023 treatment inhibited the methyltransferase activity of PRMT1 in leukemia cells from CML mice as reflected by the decreased protein level of H4R3me2a (Figure S7O, Supporting Information). CFC/replating assay showed that MS023 treatment significantly suppressed the serially plating ability of GFP^+^c‐Kit^+^ cells (Figure S7P, Supporting Information). Besides, in vivo disease reconstitution assay indicated that the recipient mice which received GFP^+^c‐Kit^+^ cells sorted from MS023‐treated primary CML mice had much lower leukemia burden (GFP^+^ cells; Figure S7Q, Supporting Information) and prolonged survival (median survival: Vehicle vs MS023 was 21 days vs not reached after monitoring for 60 days; Figure S7R, Supporting Information). These results suggest that pharmacological inhibition of PRMT1 impairs the self‐renewal of LSCs in CML mice.

### 2.7 MS023 is Minimally Detrimental to Normal Hematopoiesis in Adult Mice

Previous studies have demonstrated that *Prmt1* deletion results in severe hematopoietic phenotype. To explore the effect of MS023 on normal hematopoiesis, we treated 8‐week‐old adult C57BL/6 mice with MS023 for 2 weeks (Figure S8A, Supporting Information). The results showed that the body weight and the blood cell counts in PB, including red blood cells (RBC), white blood cells (WBC), platelets, monocytes, lymphocytes, neutrophils, and hemoglobin (HGB) did not differ between vehicle and MS023 treated adult mice (Figure S8B–I, Supporting Information). Similarly, MS023 treatment did not alter the spleen size and weight, total cell number in two legs and spleen, as well as the percentages of myeloid (Mac1^+^Gr1^+^) cells and HSPCs, including LSK cells and CMP cells in BM and spleen of adult mice as compared with vehicle treatment (Figure S8J–P, Supporting Information). These results suggest that MS023 treatment elicits minimal toxicity to normal hematopoiesis in adult mice.

Furthermore, to investigate the direct impact of MS023 on normal CD34^+^ cell engraftment, we transplanted normal CD34^+^ cells sorted from peripheral blood mononuclear cells (PBMCs) of healthy donors into NOD/ShiLtJGpt‐*Prkdc* ^em26Cd52^ *Il2*rg^em26Cd22^/Gpt (NCG) mice via tail vein injection. At week 10 post‐transplantation, the NCG mice were treated with MS023 for 2 weeks. The body weight of NCG mice was monitored and the human‐derived cells in the BM of NCG mice were detected by flow cytometry (Figure S9A, Supporting Information). The results showed that the body weight and the proportions of human CD45^+^ and CD45^+^CD34^+^ cells did not differ between vehicle and MS023 treated NCG mice (Figure S9B–F, Supporting Information). In addition, MS023 treatment did not impair the serially plating ability of normal CD34^+^CD38^−^ cells (Figure S9G, Supporting Information). These data indicate that MS023 has minimal effect on the engraftment and the self‐renewal of normal CD34^+^ cells.

### 2.8 PRMT1 Regulates the Expression of RPL29 in LSCs

**Figure 4:**
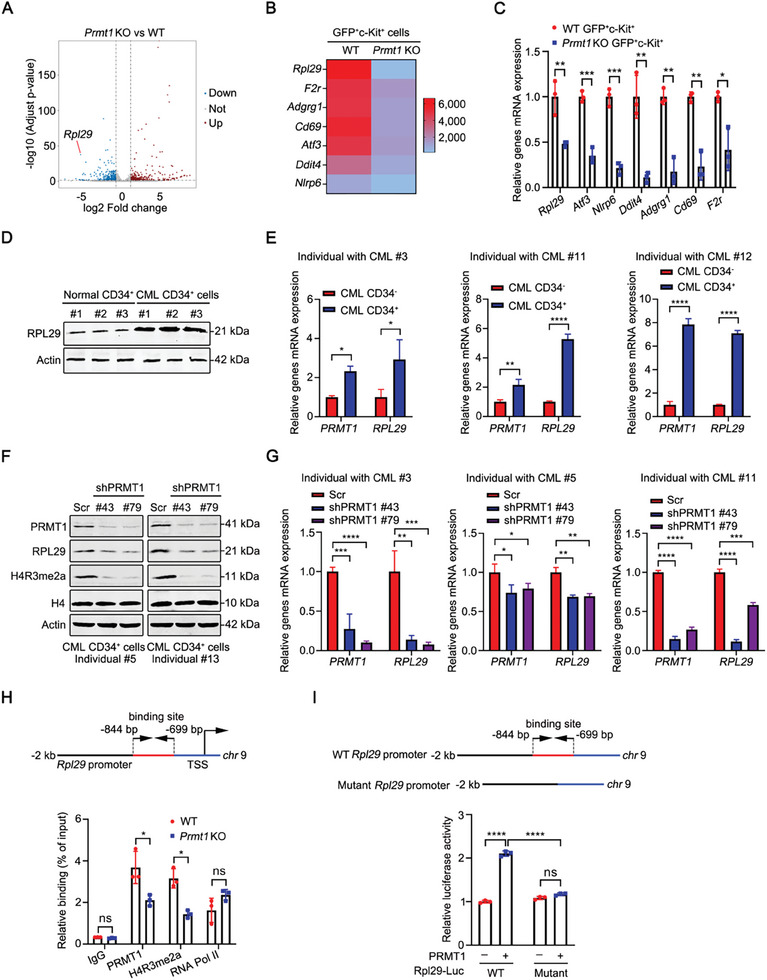
PRMT1 promotes the expression of RPL29 in LSCs. A) Volcano plot showing 335 up‐ and 343 down‐regulated genes from RNA‐seq analysis of *Prmt1* KO GFP^+^c‐Kit^+^ cells compared with WT GFP^+^c‐Kit^+^ cells sorted from primary CML mice. B) Heatmap of the downregulated genes from RNA‐seq analysis. C) qRT‐PCR analysis of the mRNA expression of *Rpl29, Atf3, Nlrp6, Ddit4, Adgrg1, Cd69* and *F2r* in GFP^+^c‐Kit^+^ cells sorted from WT or *Prmt1* KO CML mice (*n =* 3). D) Western blotting analysis of the protein level of RPL29 in CML CD34^+^ cells (*n =* 3) and normal CD34^+^ cells (*n =* 3). E) qRT‐PCR analysis of the mRNA expression of *PRMT1* and *RPL29* in CD34^+^ cells and CD34^−^ cells from the same individual with CML (*n =* 3). F, G) *PRMT1* knockdown decreased the expression of RPL29 in CML CD34^+^ cells. Western blotting analysis of the protein levels of PRMT1 and RPL29 in CML CD34^+^ cells (*n =* 2) with *PRMT1* knockdown (F). qRT‐PCR analysis of the mRNA expression of *PRMT1* and *RPL29* in CML CD34^+^ cells (*n =* 3) with *PRMT1* knockdown (G). H) The recruitment of PRMT1 and H4R3me2a at *Rpl29* gene promoter in leukemia cells from WT or *Prmt1* KO CML mice was examined by chromatin immunoprecipitation (ChIP) assay. Anti‐IgG and anti‐RNA Polymerase (Pol) II were negative and positive controls, respectively. I) PRMT1 enhanced the transcriptional activity of *Rpl29*. 293T cells were transfected with the reporter constructs encoding *Rpl29* promoter (WT *Rpl29*‐Luc) or PRMT1‐binding site deleted *Rpl29* promoter (Mutant *Rpl29*‐Luc) and pEFRenilla‐Luc together with pCDH‐*PRMT1* construct. *Rpl29* promoter activities were detected by dual‐luciferase reporter assay. Data are represented as means ± SEM. ^*^ *p* < 0.05, ^**^ *p* < 0.01, ^***^ *p* < 0.001, ^****^ *p* < 0.0001; ns, not significant, by student's *t* test (C, E and H) or one‐way ANOVA with Tukey's test (G and I).

To further explore the underlying mechanism by which PRMT1 regulates LSCs, we performed RNA sequencing (RNA‐seq) analysis by using GFP^+^c‐Kit^+^ cells sorted from BM of WT or *Prmt1* KO CML mice. The results showed that 343 and 335 genes were significantly downregulated or upregulated in *Prmt1* KO GFP^+^c‐Kit^+^ cells (**Figure** **4**A). The 7 downregulated genes listed in Figure 4B were confirmed by qRT‐PCR analysis (Figure 4C). Given that ribosomal protein L29 (*Rpl29*) ranks first within the 7 downregulated genes, we considered RPL29 as the candidate target gene of PRMT1 in LSCs. As expected, we found that the protein level of RPL29 was increased in CML CD34^+^ cells as compared with normal CD34^+^ cells (Figure 4D). We further compared the expression of RPL29 in CD34^+^ cells with CD34^−^ cells from the same individuals with CML. Our results indicated that the mRNA expression of *PRMT1* and *RPL29* was significantly increased in CML CD34^+^ cells relative to CD34^−^ cells (Figure 4E).

We next determined whether and how PRMT1 regulates RPL29 expression. *PRMT1* knockdown obviously reduced the expression of RPL29 and H4R3me2a in CML CD34^+^ cells (Figure 4F,G), suggesting that PRMT1 may induce RPL29 expression by catalyzing the methylation of H4R3 at the gene promoter of RPL29. Furthermore, we performed a chromatin immunoprecipitation (ChIP) assay to examine whether PRMT1 binds to the promoter of the *Rpl29* gene. The results showed significantly decreased enrichment of PRMT1 and H4R3me2a at the gene promoter region of *Rpl29* in leukemia cells from the *Prmt1* KO versus WT CML mice (Figure 4H). To test whether mutagenesis of the PRMT1‐binding site in *RPL29*’s promoter impairs its expression, we generated and transfected the reporter constructs encoding *Rpl29* promoter (WT *Rpl29*‐Luc) or PRMT1‐binding site deleted *Rpl29* promoter (Mutant *Rpl29*‐Luc) and pEFRenilla‐Luc together with pCDH‐*PRMT1* construct in 293T cells. *Rpl29* promoter activities were detected by dual‐luciferase reporter assay. The results showed that PRMT1 enhanced *Rpl29* but not the PRMT1‐binding site deleted *Rpl29* promoter activity (Figure 4I).

Overall, these results demonstrate that PRMT1 promotes *RPL29* gene transcription via catalyzing H4R3me2a at its gene promoter in CML LSCs.

### 2.9 RPL29 is Critical for the Survival and Serially Plating Abilities of CML LSCs

**Figure 5:**
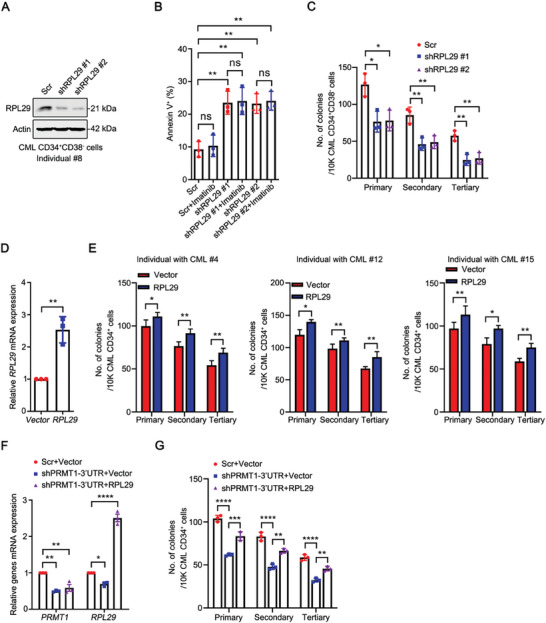
RPL29 promotes the survival and serially plating abilities of human primary CML LSCs. A, B) *RPL29* knockdown induced apoptosis in CML LSCs. The primary CML CD34^+^CD38^−^ cells (*n =* 3) were transduced with Scr, sh*RPL29* #1, or sh*RPL29* #2 lentivirus for 48 h, and then treated ± imatinib (2.5 µM) for 24 h. The apoptosis of CML CD34^+^CD38^−^ cells was detected by flow cytometry after staining with Annexin V‐FITC and PI. RPL29 knockdown was confirmed by Western blotting assay (A). Quantitative results for apoptotic cells (Annexin V^+^) were shown (B). C) Knockdown of *RPL29* inhibited the serially plating capacity of CML LSCs. The viable CML CD34^+^CD38^−^ cells (*n =* 3) with *RPL29* knockdown were seeded in methylcellulose medium (MethoCult H4434) for 3 rounds of CFC/replating assay. D, E) Overexpression of *RPL29* enhanced the serially plating capacity of CML CD34^+^ cells. CML CD34^+^ cells (*n =* 3) were transduced with vector or *RPL29* lentivirus, and then 3 rounds of CFC/replating assay was performed. The overexpression of *RPL29* was examined by qRT‐PCR analysis (D). The quantitative results for CFC/replating assay were shown (E). F, G) Restoration of *RPL29* rescued the *PRMT1* knockdown‐mediated inhibition of the serially plating ability of CML CD34^+^ cells. The primary CML CD34^+^ cells (*n =* 3) were transduced with sh*PRMT1*‐3′UTR and *RPL29* lentivirus. The mRNA expression of *PRMT1* and *RPL29* were examined by qRT‐PCR analysis (F). The quantitative results of CFC/replating assay were shown (G). Data are represented as means ± SEM. ^*^ *p* < 0.05, ^**^ *p* < 0.01, ^***^ *p* < 0.001, ^****^ *p* < 0.0001, ns, not significant, by one‐way ANOVA with Tukey's test (B, C, F and G) or student's *t* test (D and E).

To investigate the role of RPL29 in human CML LSCs, we knocked down the expression of *RPL29* by using two independent shRNAs (**Figure** **5**A). Silencing *RPL29* dramatically induced apoptosis in CML CD34^+^CD38^−^ cells (Figure 5B), without an additive or synergistic effect when co‐treated with imatinib. This may be due to the compensatory effects of other RPLs to impact the survival of CML LSCs. Moreover, *RPL29* knockdown inhibited the serially plating capacity of CML CD34^+^CD38^−^ cells (Figure 5C). Conversely, overexpressed *RPL29* significantly enhanced the serially plating ability of CML CD34^+^ cells (Figure 5D,E). To determine whether RPL29 is a functional mediator in PRMT1 regulation of LSCs, we restored *RPL29* expression in *PRMT1*‐silenced CML CD34^+^ cells (Figure 5F) and performed a CFC/replating assay. The results revealed that restoration of *RPL29* rescued the *PRMT1* knockdown‐mediated serially plating capacity inhibition of CML CD34^+^ cells (Figure 5G).

Overall, these results indicate that RPL29 plays an important role in promoting the survival and serially plating abilities of CML LSCs.

### 2.10 RPL29 is Responsible for the Development of BCR‐ABL‐Driven CML Mice

To explore the role of RPL29 in CML development, we silenced *Rpl29* in BM and spleen cells from primary WT CML mice by using two independent shRNAs and transplanted these cells into secondary recipient mice to induce CML (Figure S10A, Supporting Information). RPL29 knockdown in splenic leukemia cells was confirmed by Western blotting analysis (Figure S10B, Supporting Information). Silencing *Rpl29* significantly prolonged the survival (median survival: Scr vs sh*Rpl29* #1 vs sh*Rpl29* #2 was 16 days vs not reached after monitoring for 50 days; Figure S10C, Supporting Information), obiviously alleviated the splenomegaly and the spleen weight of CML mice (Figure S10D,E, Supporting Information). Flow cytometry analysis showed that knockdown of *Rpl29* significantly decreased the leukemia burden and eliminated LSPCs in BM and spleen of secondary CML mice (Figure S10F–K, Supporting Information). In addition, *Rpl29* knockdown dramatically inhibited the serially plating ability of LSCs sorted from secondary CML mice (Figure S10L, Supporting Information).

Conversely, we investigate the effect of RPL29 overexpression on CML development. We transduced BM and spleen cells from primary WT CML mice with pCDH‐vector or pCDH‐*Rpl29* lentivirus and transplanted these cells into secondary recipient mice to induce CML (Figure S10M, Supporting Information). RPL29 overexpression in splenic leukemia cells was confirmed by Western blotting analysis (Figure S10N, Supporting Information). The results demonstrated that overexpression of *Rpl29* shortened survival (median survival: Vector vs *Rpl29* was 29 vs 18 days), worsened the splenomegaly and spleen weight, and increased the leukemia burden as well as the percentages of LSPCs in BM and spleen of secondary CML mice (Figure S10O–W, Supporting Information).

Taken together, these data indicate that RPL29 is critical for LSCs and the progression of CML.

### 2.11 Restoration of *Rpl29* Rescues the *Prmt1* Deletion‐Mediated Elimination of LSCs in CML Mice

**Figure 6:**
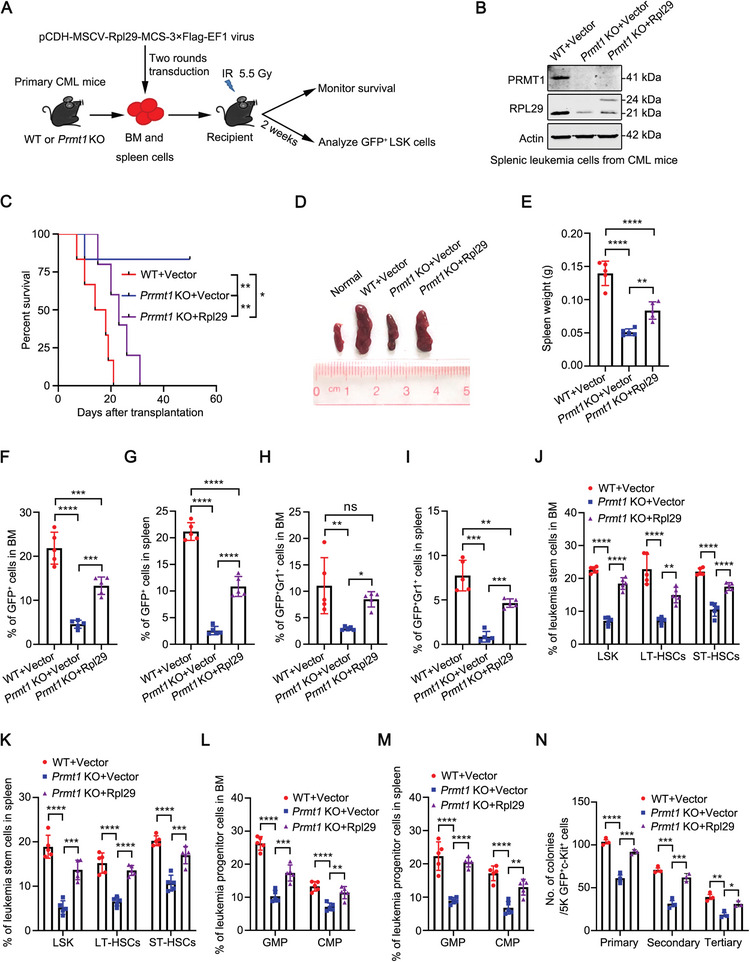
Re‐introduction of *Rpl29* rescues the *Prmt1* KO‐mediated elimination of LSCs in CML mice. A) Experimental strategy to determine the role of RPL29 in PRMT1 regulation of LSCs. BM and spleen cells from the primary WT or *Prmt1* KO CML mice were transduced with lentivirus (Vector or *Rpl29*) and transplanted into the secondary recipients to induce CML. B) Western blotting analysis of the protein levels of PRMT1 and RPL29 in splenic leukemia cells from the secondary CML mice. C) Kaplan–Meier survival curves. *n =* 5–6 mice per group. D) Representative photograph of the spleen from each group. E) The weight of spleens was shown. *n =* 5 mice per group. F–M) Restoration of *Rpl29* rescued the reduction of LSCs in CML mice mediated by *Prmt1* KO. The percentages of GFP^+^ cells (F, G), GFP^+^Gr1^+^ cells (H, I), GFP^+^LSK cells, GFP^+^LT‐HSCs and GFP^+^ST‐HSCs (J, K), as well as GFP^+^GMP and GFP^+^CMP cells (L, M) in BM and spleen were analyzed by flow cytometry. *n =* 5 mice per group. N) The GFP^+^c‐Kit^+^ cells sorted from the indicated secondary CML mice were subjected to CFC/replating assay. Data are represented as means ± SEM. ^*^ *p* < 0.05, ^**^ *p* < 0.01, ^***^ *p* < 0.001, ^****^ *p* < 0.0001, ns, not significant, by one‐way ANOVA with Tukey's test (E‐N) or log‐rank test (C).

To determine whether RPL29 is a functional mediator in PRMT1 regulation of LSCs in vivo, we transduced BM and spleen cells from primary WT or *Prmt1* KO CML mice with pCDH‐vector or pCDH‐*Rpl29* lentivirus and transplanted these cells into secondary recipient mice to induce CML (**Figure** **6**A). The successful overexpression of RPL29 in splenic leukemia cells from secondary CML mice was confirmed by Western blotting analysis (Figure 6B). The recipient mice that received *Prmt1* KO leukemia cells overexpressed *Rpl29* exhibited shorter survival (median survival: *Prmt1* KO + *Rpl29* vs *Prmt1* KO+Vector was 23 days vs not reached after monitoring for 50 days), more prominent splenomegaly and spleen weight than those that received *Prmt1* KO leukemia cells overexpressed empty vector (Figure 6C–E). Consistently, flow cytometry analysis showed that restoration of *Rpl29* rescued the *Prmt1* deletion‐mediated reductions of the leukemia burden and proportions of LSPCs in secondary CML mice (Figure 6F–M). In addition, re‐introduction with *Rpl29* rescued the *Prmt1* deletion‐mediated decrease of serially plating ability of LSCs (Figure 6N).

Taken together, these results demonstrate that ectopic overexpression of *Rpl29* rescues the *Prmt1* loss‐mediated elimination of LSCs in CML mice.

### 2.12 PRMT1 Regulates the Global Protein Synthesis Rates via RPL29 in CML LSCs

**Figure 7:**
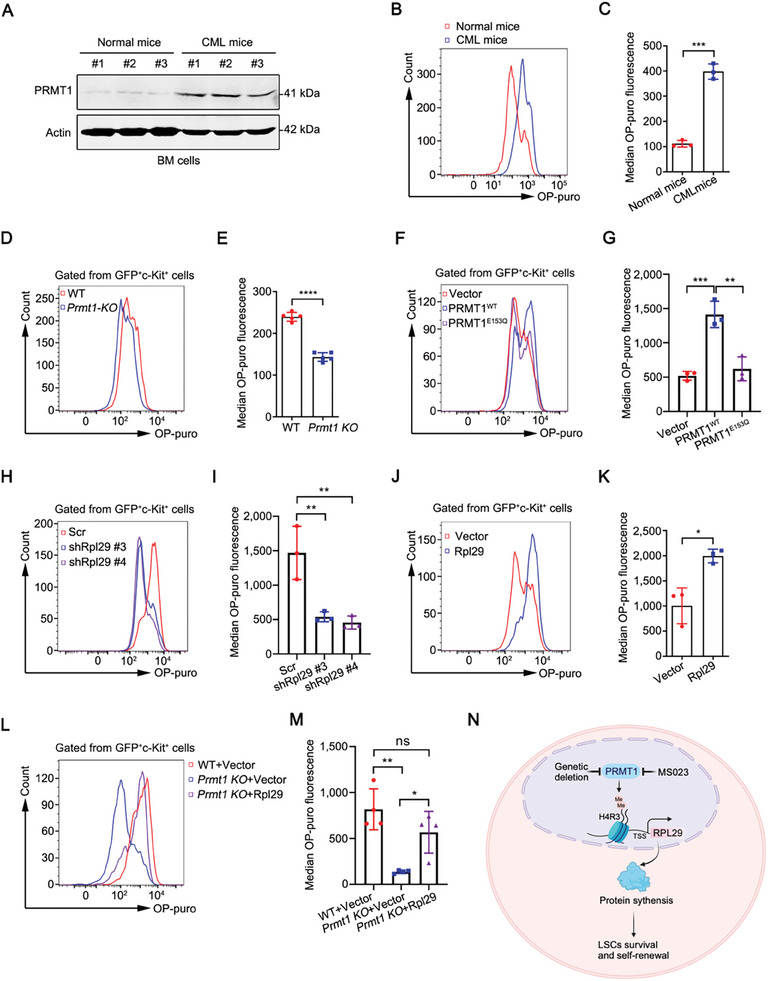
PRMT1 augments the global protein synthesis via RPL29 in CML LSCs. A–C) In vitro O‐propargyl‐puromycin (OP‐Puro) incorporation assay in BM cells from CML or normal mice (*n =* 3). Western blotting analysis of the protein level of PRMT1 in BM cells from CML or normal mice (A). Representative flow cytometry histograms (B) and statistical results of median OP‐Puro fluorescence in BM cells (C) were shown. D, E) In vitro OP‐Puro incorporation assay in LSPCs from WT or *Prmt1* KO CML mice. Representative flow cytometry histograms (D) and statistical results of median OP‐Puro fluorescence in gated GFP^+^c‐Kit^+^ cells (E) were shown. *n =* 5 mice per group. F, G) The methyltransferase activity of PRMT1 was responsible for the augmentation of global protein synthesis in GFP^+^c‐Kit^+^ cells. The leukemia cells from primary WT CML mice were transduced with vector, *PRMT1^WT^* or *PRMT1^E153Q^* lentivirus, and then the global protein synthesis in gated GFP^+^c‐Kit^+^ cells was detected by OP‐Puro incorporation assay. Representative flow cytometry histograms (F) and statistical results of median OP‐Puro fluorescence (G) were shown. *n =* 3 mice per group. H‐K) RPL29 promoted the global protein synthesis. The leukemia cells from primary WT CML mice were transduced with Scr, sh*Rpl29* (#3 or #4), pCDH‐vector or pCDH‐*Rpl29* lentivirus, respectively. The protein synthesis was detected by OP‐Puro incorporation assay. Representative flow cytometry histograms (H and J) and statistical results of median OP‐Puro fluorescence (I and K) were shown. *n =* 3 mice per group. L, M) Overexpression of *Rpl29* rescued the decreased protein synthesis mediated by *Prmt1* loss. Representative flow cytometry histograms (L) and statistical results of median OP‐Puro fluorescence (M) were shown. *n =* 4 mice per group. N) Proposed working model for PRMT1‐RPL29 mediated the protein synthesis in sustaining self‐renewal of LSCs. Data are represented as means ± SEM. ^*^ *p* < 0.05, ^**^ *p* < 0.01, ^***^ *p* < 0.001, ^****^ *p* < 0.0001, ns, not significant, by student's *t* test (C, E and K) or one‐way ANOVA with Tukey's test (G, I and M).

RPL29, as a component of the large ribosomal subunit, plays an important role in the regulation of global protein synthesis.^[^ ^31^ ^]^ We hypothesized that PRMT1 promotes the self‐renewal of LSCs via RPL29‐mediated global protein synthesis. To address this idea, we examined the global protein synthesis by using an O‐propargyl‐puromycin (OP‐Puro) incorporation assay.^[^ ^32^ ^]^ After confirming that PRMT1 was highly expressed in CML cells (**Figure** **7**A), we detected the global protein synthesis rates in BM cells from CML mice and normal mice. The results showed that the global protein synthesis rates were dramatically increased in CML cells as compared to normal cells (Figure 7B,C), indicating that PRMT1 augments the global protein synthesis rates in CML cells. Conversely, the loss of *Prmt1* reduced the global protein synthesis rates in GFP^+^c‐Kit^+^ cells from CML mice (Figure 7D,E). Furthermore, overexpression of *PRMT1^WT^* but not *PRMT1^E153Q^* significantly increased the global protein synthesis rates in gated GFP^+^c‐Kit^+^ cells (Figure 7F,G), suggesting that the methyltransferase activity of PRMT1 is essential for the augmentation of the global protein synthesis rates in LSCs. Similarly, *Rpl29* knockdown significantly reduced and *Rpl29* overexpression significantly increased the global protein synthesis rates in gated GFP^+^c‐Kit^+^ cells (Figure 7H–K). Furthermore, ectopic expression of *Rpl29* remarkably reversed the decreased global protein synthesis rates mediated by *Prmt1* KO in gated GFP^+^c‐Kit^+^ cells (Figure 7L,M). These findings suggest that PRMT1 may regulate the global protein synthesis rates via RPL29 in CML LSCs.

## 3 Discussion

The persistence of LSCs may drive disease relapse and progression which remains a big challenge and an unmet clinical need in patients with CML. Therefore, understanding the biology of LSCs and identifying the druggable target(s) are urgently needed. In the present study, we validated PRMT1 as a promising therapeutic target in CML LSCs. Targeting PRMT1 inhibited the survival and serially plating abilities of human primary CML LSCs. Genetic loss of *Prmt1* or pharmacological inhibition of PRMT1 remarkably blocked leukemia development, eliminated LSCs, and impaired the self‐renewal of LSCs in CML mice. Mechanistically, PRMT1 enhanced the gene transcription of RPL29 by catalyzing H4R3me2a at its gene promoter. RPL29 was a functional mediator in the PRMT1 regulation of LSCs. PRMT1 augmented the global protein synthesis via RPL29 in CML LSCs (proposed working model, Figure 7N).

PRMT1, the most predominant arginine methyltransferase, plays a critical role in tumorigenesis, especially in hematological malignancies, including leukemia and multiple myeloma (MM). It has been reported that PRMT1 promotes FLT3‐ITD^+^ AML cell growth through methylating FLT3‐ITD protein. Combined treatment of type I PRMTs inhibitor MS023 with FLT3 TKI AC220 eliminates FLT3‐ITD^+^ AML cells in a patient‐derived xenograft mouse model.^[^ ^25^ ^]^ PRMT1 interacts with AE9a and transcriptionally activates the expression of AE9a‐activated genes via promoting the enrichment of H4R3me2a and H3 Lys9/14 acetylation (H3K9/14Ac) at the active promoters, leading to the leukemia development.^[^ ^33^ ^]^ Another study has revealed that PRMT1 can block megakaryocyte differentiation in acute megakaryocytic leukemia via directly methylating the RNA binding protein RBM15.^[^ ^34^ ^]^ In addition, PRMT1 is highly expressed in myelodysplastic syndrome patients. PRMT1‐mediated methylation of dual‐specificity phosphatase 4 triggers its ubiquitylation and degradation, leading to activation of p38 kinase and blockade of megakaryocyte differentiation.^[^ ^35^ ^]^ Recent studies have demonstrated that PRMT1 is highly expressed in MM patients and promotes MM progression in vitro and in vivo, suggesting that PRMT1 may be a potential therapeutic vulnerability in MM. PRMT1 enzymatic inhibition can increase the sensitivity of MM cells to proteasome inhibitor bortezomib.^[^ ^24^, ^36^ ^]^ In addition, it has been shown that PRMT1‐mediated arginine methylation regulates muscle stem cell fate and epithelial cell stemness.^[^ ^37^, ^38^ ^]^ However, the role of PRMT1 in LSCs is largely unknown. In this study, we found that the highly expressed PRMT1 promoted the survival and serially plating features of human primary CML LSCs. Genetic deletion of *Prmt1* delayed leukemia development, eliminated LSCs, and impaired the self‐renewal of LSCs in CML mice. Consistent with the previous reports, these results suggested that PRMT1 serves as an oncogenic protein in LSCs. To our knowledge, this is the first study to validate the important role of PRMT1 in leukemogenesis and the maintenance of LSCs in CML. Our results showed that knockdown of *PRMT1* expression or pharmacological inhibition of PRMT1 activity increased Annexin V^+^ cells, which suggests that PRMT1 inhibition may induce apoptosis in CML LSCs. It was reported that PRMT1 inhibition promotes ferroptosis or necroptosis in cancer cells. For instance, a previous study has shown that targeting PRMT1 causes ferroptosis by upregulating acyl‐CoA synthetase long‐chain family member 1 (ACSL1) in AML cells.^[^ ^39^ ^]^ Besides, silencing PRMT1 increases the necrotic cell death in colon cancer cells.^[^ ^40^ ^]^ Thus, other forms of cell death induced by PRMT1 inhibition in CML LSCs should be further investigated.

Previous studies have demonstrated that PRMT1 is required for the differentiation and self‐renewal of HSPCs. *Prmt1* KO leads to anemia and leukopenia and affects multilineage differentiation and repopulation capacity of HSCs in adult mice.^[^ ^41^ ^]^ Our results showed that *Prmt1* deletion effectively eliminated LSCs and suppressed the self‐renewal of LSCs in CML mice. Pharmacological inhibition of PRMT1 by MS023 dramatically reduced the proportions of LSPCs in CML mice. Impressively, MS023 was minimally detrimental to normal hematopoiesis in adult mice as well as the engraftment and the self‐renewal of normal CD34^+^ cells. PRMT1 may serve as a promising therapeutic target for CML. We know that MS023 is a type I PRMTs inhibitor but not a specific PRMT1 inhibitor. However, MS023 has been commonly used to inhibit PRMT1 methyltransferase activity in the previously published studies.^[^ ^25^, ^42^ ^]^ MS023 has been demonstrated to inhibit multiple types I PRMTs (1, 3, 4, 6, 8) to a similar degree^[^ ^29^ ^]^ and multiple PRMTs seem to be functionally important in leukemia. Therefore, MS023 could have effects on leukemia that are not mediated by PRMT1. The specific PRMT1 inhibitors should be designed and their effects on CML LSCs should be further investigated in the future.

Epigenetic regulators play critical roles in leukemia development and the function of LSCs. Our previous study showed that PRMT5 activates the Wnt/β‐catenin signaling pathway by transcriptional regulation of dishevelled homolog 3 and promotes the self‐renewal of CML LSCs.^[^ ^14^ ^]^ We also demonstrated that PRMT7 catalyzes the arginine symmetric dimethylation of histone H2A at the transcriptional repressor GATA binding 1 promoter and suppresses its gene transcription, increases glycine decarboxylase expression, and accelerates glycine metabolism in CML LSCs.^[^ ^15^ ^]^ However, we found that PRMT1 knockdown did not affect the Wnt/β‐catenin signaling pathway (Figure S11A, Supporting information) and alter the protein levels of PRMT5 and PRMT7 in CML CD34^+^ cells (Figure S11B, Supporting information). PRMT1, PRMT5, and PRMT7 belonging to different types of arginine methyltransferases prompted us to hypothesize that they may regulate LSCs through distinct mechanisms. In the present study, we identified RPL29 as the downstream target gene of PRMT1 by analyzing our RNA‐seq data. PRMT1 enhanced *RPL29* gene transcription via catalyzing H4R3me2a at its gene promoter. Of interest, RPL29 was identified as an oncoprotein in various cancers, such as pancreatic cancer, lung cancer, and colon cancer.^[^ ^43^, ^44^, ^45^ ^]^ Consistent with the previous reports, our results demonstrated that RPL29 plays an important role in sustaining the survival and self‐renewal characteristics of primary human CML LSCs. *Rpl29* knockdown eliminated LSCs and extended the survival of CML mice while *Rpl29* overexpression accelerated leukemia development. Restoration of *Rpl29* rescued the *Prmt1* deletion‐mediated elimination of LSCs in CML mice, suggesting that RPL29 is a functional mediator in PRMT1 regulation of LSCs.

RPL29, a key ribosomal protein in the ribosome checkpoint pathway, is ubiquitously expressed in all types of cells. As an accessory protein, RPL29 plays an active role in the modulation of translation efficiency and global protein synthesis. Rpl29 null embryonic fibroblasts display reduced global protein synthesis.^[^ ^31^ ^]^ However, RPL29 methylation at lysine 5 mediated by the lysine methyltransferase Set7/9 does not affect the global protein synthesis but affects its subcellular localization.^[^ ^46^ ^]^ It has been shown that HSCs have very low protein synthesis rates, while LSCs undergo a massive clonal expansion during leukemogenesis to maintain self‐renewal and rely on increasing protein synthesis. Rapamycin, the mTORC1 inhibitor, can inhibit protein synthesis and decrease LSCs frequency in AML‐ETO9a‐driven leukemia (BioRxiv preprint doi: https://doi.org/10.1101/2023.02.21.529419). Our data revealed that PRMT1 promotes the self‐renewal of LSCs via RPL29‐mediated global protein synthesis.

In conclusion, our results revealed that PRMT1 was required for the function of human primary CML LSCs, the development of leukemia, and the disease reconstitution ability of LSCs in CML mice. RPL29 was identified as the key functional mediator in PRMT1 regulation of LSCs. PRMT1 augmented the global protein synthesis via H4R3me2a‐mediated transcriptional activation of RPL29 in CML LSCs, allowing for their self‐renewal capacity. This is the first report to link histone arginine methylation with protein synthesis in the maintenance of LSCs. PRMT1 may represent a therapeutic vulnerability in patients with CML.

## 4 Experimental Section

### DNA Constructs

MSCV‐*BCR‐ABL*‐IRES‐GFP and MSCV‐*T315I‐BCR‐ABL*‐IRES‐GFP constructs were described previously.^[^ ^15^, ^16^, ^47^, ^48^ ^]^ MSCV‐*BCR‐ABL‐iCre*‐IRES‐GFP construct was kindly provided by Dr. Haojian Zhang (Wuhan University, Wuhan, China).^[^ ^49^ ^]^ Human pCDH‐CMV‐*PRMT1^WT^*‐MCS‐Flag‐EF1, human pCDH‐CMV‐*PRMT1^E153Q^*‐MCS‐Flag‐EF1, human pCDH‐MSCV‐*RPL29*‐MCS‐3 × Flag‐EF1, murine pCDH‐MSCV‐*Rpl29*‐MCS‐3 × Flag‐EF1 constructs, human *PRMT1* shRNAs, *RPL29* shRNAs, and murine *Rpl29* shRNAs were purchased from TranSheepBio (Shanghai, China).

### Human Primary Cells

PB samples derived from CML patients or healthy adult donors were obtained from the First Affiliated Hospital of Sun Yat‐sen University, Guangdong Provincial People's Hospital (Guangdong Academy of Medical Sciences), and the First Affiliated Hospital of Jinan University, with written informed consent in accordance with the Declaration of Helsinki principles. This study was approved by the Jinan University Ethics Committee (Approval number: JNUKY‐2023‐0065). Primary CD34^+^ cells were sorted using a CD34 Microbead Kit according to the manufacturer's recommendations. CML CD34^+^CD38^−^ cells were sorted using CD38 Microbead Kit and CD34 Microbead Kit according to the manufacturer's recommendations. Briefly, the CD38^−^ cells were sorted from PBMCs isolated from CML patient samples using CD38 Microbead Kit, and then subjected to purify CD34^+^CD38^−^ cells using CD34 Microbead Kit. The human primary cells were cultured in Iscove's modified Dulbecco's medium (IMDM) containing 20% fetal bovine serum (FBS) and the following cytokines: stem cell factor (SCF, 100 ng mL^−1^), interleukin‐3 (IL‐3, 20 ng mL^−1^), IL‐6 (10 ng mL^−1^), fms‐related tyrosine kinase 3 (Flt‐3, 10 ng mL^−1^) and thrombopoietin (TPO, 100 ng mL^−1^) at 37 °C in a humidified incubator with 5% CO_2_ as previously reported.^[^ ^15^, ^16^, ^48^ ^]^ Detailed information for patients with CML was summarized in Table S2 (Supporting Information).

### Lentiviral Transduction of Primary CML CD34^+^ or CD34^+^CD38^−^ Cells

The primary CML CD34^+^ cells or CD34^+^CD38^−^ cells were resuspended in IMDM supplemented with indicated lentivirus and polybrene (8 mg mL^−1^) and centrifuged 90 min at 1500 × *g*, 32 °C for two rounds. The cells were selected by treatment with puromycin (0.5 µg mL^−1^) or G418 (200 µg mL^−1^) for 48 h.

### Measurement of Apoptosis in Primary CML CD34^+^CD38^−^ Cells

The primary CML CD34^+^CD38^−^ cells were transduced with indicated lentivirus or treated with MS023 for 48 h. The percentage of apoptotic cells (Annexin V^+^) was measured using Annexin V‐FITC apoptosis detection Kit (MERCK, Cat# APOAF) by flow cytometry (BD LSRFortessa) as previously described.^[^ ^15^, ^16^, ^48^ ^]^

### Colony‐forming Cell/Replating (CFC/replating) Assay

Primary CML CD34^+^ or CD34^+^CD38^−^ cells were transduced with indicated lentivirus or treated with MS023 for 48 h, and the viable cells were seeded in MethoCult H4434 methylcellulose medium for three rounds of the replating assay. Colonies were counted on day 10‐14 after seeding, as previously described.^[^ ^15^, ^16^, ^48^ ^]^ GFP^+^c‐Kit^+^ cells (5000 cells/well) sorted from CML mice were cultured in MethoCult M3434 methylcellulose medium for three rounds of the replating assay. Colonies were counted on day 7 after seeding.^[^ ^15^, ^47^ ^]^

### Mice

*Prmt1^fl/fl^* mice and Rosa26‐*Cre‐ER^T2^* mice in a C57BL/6 background were kindly provided by Dr. Shilai Bao (Institute of Genetics and Developmental Biology, Chinese Academy of Sciences, Beijing, China). For the *Prmt1* ^fl/fl^ mouse strain (Mouse Genome Informatics [MGI]: 4432476), exons 4 and 5 of the *Prmt1* gene were flanked by two loxP sites.^[^ ^50^ ^]^ The *Prmt1* ^fl/fl^ mice were bred with Rosa26‐*Cre‐ER^T2^* (Cre‐ER^T2^) mice ([MGI]: 3699244) harboring the *Cre‐ER^T2^* coding region under the control of Rosa26 locus^[^ ^51^, ^52^ ^]^ to generate tamoxifen‐inducible *Prmt1* global knockout mice (*Prmt1^fl/fl^*;*Cre‐ER^T2^*). *Prmt1* ^fl/fl^ mice were genotyped by PCR (forward: 5′‐GTGCTTGCCATACAAGAGATCC‐3′, reverse: 5′‐ACAGCCGAGTAGCAAGGAGG‐3′). Rosa26‐*Cre‐ER^T2^* mice were genotyped by PCR (forward: 5′‐CTCTATGACCTGCTGCTGGAG‐3′, reverse: 5′‐ACGGGAAGCAATAGCATGATAC‐3′).

C57BL/6 mice were purchased from Guangdong Medical Laboratory Animal Center (Guangzhou, China). The mice were housed in the standard specific pathogen‐free facility with a 12 h light/dark cycle and obtained standard mouse chow and water ad libitum. All mice used in this study were healthy. The animal studies were conducted under an established protocol approved by the Institutional Animal Care and Use Committee of South China University of Technology (Approval number: 2024017).

### CML Mouse Model

Donor C57BL/6 mice (male, 6–8 weeks) were treated with 5‐FU (200 mg kg^−1^) via tail vein injection for 5 days. The BM cells stimulated overnight in DMEM containing SCF (50 ng mL^−1^), IL‐3 (6 ng mL^−1^), and IL‐6 (10 ng mL^−1^) were transduced with MSCV‐*BCR‐ABL*‐IRES‐GFP, MSCV‐*T315I*‐*BCR‐ABL*‐IRES‐GFP or MSCV‐*BCR‐ABL‐iCre*‐IRES‐GFP retrovirus for two rounds (1500 × *g*, 32 °C, 90 min). The viable cells (1 × 10^6^ cells/mouse) were transplanted into sublethally irradiated (550 cGy) recipient C57BL/6 mice (female, 6–8 weeks) to induce CML.^[^ ^15^, ^47^ ^]^

To explore the effect of tamoxifen‐induced *Prmt1* KO on leukemogenesis, the donor mice (*Prmt1^fl/fl^* or *Prmt1^fl/fl^*;*Cre‐ER^T2^*) were pretreated with tamoxifen (100 mg kg^−1^, gavage) on day 1, 3, 5 and 7 before induction of CML.^[^ ^53^ ^]^ To assess the effects of *Prmt1* loss on the propagation of leukemia, BM cells from 5‐FU‐treated *Prmt1^fl/fl^*;*Cre‐ER^T2^* or *Prmt1^fl/fl^* mice were transduced with BCR‐ABL‐GFP retrovirus and transplanted them into sublethally irradiated recipients. The recipients were administered tamoxifen (100 mg kg^−1^, gavage) on days 7, 9, 11, and 13 post‐transplantation.

To determine the effect of *Rpl29* knockdown or overexpression in vivo, leukemia cells from primary WT CML mice were transduced with control shRNA (Scr) and murine *Rpl29* shRNAs (sh*Rpl29* #1 or sh*Rpl29* #2) or vector and *Rpl29* lentivirus for two rounds. The viable cells (2 × 10^6^ cells/mouse) were injected into the sublethally irradiated (550 cGy) recipient mice (female, 6–8 weeks) via tail vein injection.^[^ ^15^, ^16^, ^48^ ^]^

To assess the effect of *PRMT1* or *Rpl29* restoration in vivo, BM and spleen cells from primary CML mice (WT or *Prmt1* KO) were transduced with indicated lentivirus (vector, *PRMT1^WT^*, *PRMT1^E153Q^*) or (vector, *Rpl29*) and then transplanted into the sublethally irradiated (550 cGy) recipient mice to develop secondary CML.^[^ ^15^, ^16^, ^48^ ^]^

### Flow Cytometry Analysis of Leukemia Stem/Progenitor Cells in CML Mice

To analyze the populations of myeloid leukemia cells and LSPCs in CML mice, BM and spleen cells from CML mice were analyzed by flow cytometry after staining with the indicated antibodies. For analysis of myeloid leukemia cells, cells were stained with APC anti‐mouse Gr‐1 antibody (BD Biosciences, Cat# 553129); For analysis of leukemia stem cells, cells were stained with APC anti‐mouse Lin antibody (BD Biosciences, Cat# 558074), PE‐CF594 anti‐mouse Sca‐1 antibody (BD Biosciences, Cat# 562730), PE anti‐mouse c‐Kit antibody (BD Biosciences, Cat# 553355), PE‐Cy5 anti‐mouse CD135 antibody (eBioscience, Cat# 15‐1351‐82), PE‐Cy7 anti‐mouse CD150 antibody (eBioscience Cat# 25‐1502‐82), and APC‐Cy7 anti‐mouse CD48 antibody (BD Biosciences, Cat# 561242); For analysis of leukemia progenitor cells, cells were stained with APC anti‐mouse Lin antibody (BD Biosciences, Cat# 558074), PE‐CF594 anti‐mouse Sca‐1 antibody (BD Biosciences, Cat# 562730), APC‐H7 anti‐mouse c‐Kit antibody (BD Biosciences, Cat# 560185), PE anti‐mouse CD34 antibody (BD Biosciences, Cat# 551387), and PE‐Cy7 anti‐mouse CD16/32 antibody (eBioscience, Cat# 25‐0161‐82), as previously reported.^[^ ^15^, ^47^ ^]^

### GFP^+^c‐Kit^+^ Cell Sorting and Secondary BM Transplantation

To sort GFP^+^c‐Kit^+^ cells, BM and spleen cells from primary CML mice were incubated with PE anti‐mouse c‐Kit antibody (BD Biosciences, Cat# 553355) for 30 min and then sorted by flow cytometry (BD FACSAria II).

To explore the in vivo effect of *Prmt1* KO on the self‐renewal of LSCs, GFP^+^c‐Kit^+^ cells (2 × 10^5^ cells/mouse) sorted from primary WT or *Prmt1* KO CML mice were transplanted into sublethally irradiated (550 cGy) recipient C57BL/6 mice. The percentage of GFP^+^ cells in the PB of recipients was detected by flow cytometry on days 8, 14, and 21 and the survival of recipients was monitored.

### NOD/ShiLtJGpt‐Prkdc^em26Cd52^Il2rg^e^ ^m26Cd22^/Gpt (NCG) Mouse Model

Primary normal CD34^+^ cells (1.5 × 10^6^/mouse) purified from PBMCs of healthy donors using a CD34 Microbead Kit were transplanted into sublethally irradiated (100 cGy) 6‐8‐week‐old female NCG mice (GemPharmatech, Jiangsu, China) via tail vein injection. At week 10 post‐transplantation, the mice were administered vehicle or MS023 (80 mg kg^−1^/day, i.p.) for 2 weeks. BM cells collected from the mice were analyzed by flow cytometry (BD LSRFortessa) after staining with the antibodies anti‐human CD45‐APC‐Cy7 (BD Biosciences, Cat# 557833), anti‐human CD34‐FITC (eBioscience, Cat# 11‐0349‐42).

### In Vivo Limiting Dilution Assay

A serial density of BM cells from WT or *Prmt1* KO CML mice (2 × 10^6^, 1 × 10^6^, 5 × 10^5^ cells/mouse) were mixed with normal BM cells (2 × 10^5^ cells/mouse) and transplanted into the sublethally irradiated recipient mice. GFP^+^ cells in PB were detected at week 16 post‐transplantation by flow cytometry. The recipient was considered as a CML mouse when the percentage of GFP^+^ cells > 0.5%. The frequency of LSCs was calculated as previously reported.^[^ ^15^, ^16^, ^48^ ^]^

### RNA Sequencing Analysis

Total mRNA was extracted from GFP^+^c‐Kit^+^ cells sorted from the primary WT or *Prmt1*‐deleted CML mice by using Trizol reagent and subjected to RNA‐sequencing analysis. Sequencing libraries were generated by NEBNext UltraTM RNA Library Prep Kit for Illumina (NEB, USA) according to the manufacturer's instructions and index codes were added to attribute sequences to each sample. Differentially expressed genes were screened according to the following criteria: Fold change ≥ 2 and FDR < 0.05 as previously described.^[^ ^15^, ^16^, ^48^ ^]^

### Chromatin Immunoprecipitation (ChIP) Assay

ChIP assays were examined using an EZ‐ChIP Kit as previously described.^[^ ^15^ ^]^ Briefly, leukemia cells from WT or *Prmt1* KO CML mice were crosslinked with 37% formaldehyde at room temperature for 10 min, then glycine was added to quench the untreated formaldehyde. Cells were washed with cold PBS and lysed in SDS lysis buffer with a protease inhibitor cocktail. The samples were sonicated to shear the crosslinked DNA to 200–1000 bp. The supernatants were collected after centrifuging (10 000 × g, 4 °C, 10 min) and immunoprecipitated with anti‐PRMT1, anti‐RNA Pol II, anti‐H4R3me2a, or anti‐IgG antibodies at 4 °C with rotation overnight. The pellets were harvested, and washed, and then protein‐DNA complexes were eluted and reversed by incubation at 65 °C with proteinase K. Immunoprecipitated DNA was purified by using spin columns and amplified by qRT‐PCR. The primers are as follows: *Rpl29* promoter: forward 5′‐GCAACCACATGATGGCTCACA‐3′, reverse 5′‐ AGAGTGGCAGTTTCGTGGT‐3′.

### Dual‐Luciferase Reporter Assay

The *Rpl29* promoter region or PRMT1 binding site deleted the *Rpl29* promoter region and was cloned into the pGL4.10 vector (TranSheepBio, Shanghai, China). 293T cells were transfected with the reporter constructs encoding *Rpl29* promoter (WT *Rpl29*‐Luc) or PRMT1‐binding site deleted *Rpl29* promoter (Mutant *Rpl29*‐Luc) and pEFRenilla‐Luc together with pCDH‐*PRMT1* construct for 48 h. *Rpl29* promoter activities were detected by a dual‐luciferase reporter assay Kit (Beyotime, Cat# RG027).

### In Vitro Protein Synthesis Assay

In vitro protein synthesis assay was performed as previously reported.^[^ ^32^ ^]^ Briefly, leukemia cells (GFP^+^) obtained from the BM of WT or *Prmt1* KO CML mice were cultured in IMEM containing 20% FBS (TransGen Biotech) and the following cytokines: SCF (50 ng mL^−1^), IL‐3 (6 ng mL^−1^), and IL‐6 (10 ng mL^−1^). O‐Propargyl‐puromycin (OP‐Puro, 20 µM) was immediately added into the medium and incubated at 37 °C in a humidified incubator with 5% CO_2_ for 1 h. Cells were then incubated with PE anti‐mouse c‐Kit antibody (BD Biosciences, Cat# 553355) for 1 h to identify CML LSCs (GFP^+^c‐Kit^+^ cells), followed by washing with PBS containing 2% BSA and fixation/permeabilization using the fixation/permeabilization Kit (eBioscience, Cat# 00‐5523‐00). Cells were pelleted and resuspended in the buffer from the Click‐iT cell reaction buffer Kit (Thermo Fisher Scientific, Cat# C10269). The azide‐alkyne cycloaddition was performed after adding Alexa Fluor 647 azide (Thermo Fisher Scientific, Cat# A10277, 5 µM) into the cells. The cells were washed once with PBS containing 2% BSA after reaction for 30 min at RT, then resuspended in PBS containing 0.5% BSA and analyzed by flow cytometry. “Median OP‐Puro fluorescence” reflected absolute fluorescence values for each cell sample obtained from individual CML mouse.

### Statistical Analysis

Data were presented as means ± SEM. Statistical analyses were performed with GraphPad Prism 8.0 (GraphPad, San Diego, CA). Two‐tailed Student's *t*‐test was used to calculate the *p* values for comparisons between two groups, and one‐way ANOVA, *post hoc* intergroup comparisons, and Tukey's test were used to calculate the *p* values for comparisons among multiple groups. Log‐rank test was used to calculate the *p* values for Kaplan–Meier survival curves. Sample size (n) for statistical analyses was shown in related figure legends. *p* < 0.05 was considered statistically significant.

## Conflict of Interest

The authors declare no conflict of interest.

## Author Contributions

M.Z., Y.H., C.L., and Y.L.J. designed the research. M.Z., Y.H., P.X., S.Y.L., C.D., and X.Y.L performed the experiments and analyzed and interpreted the data. W.Y.Z. provided CML patient samples and information. S.L.B. provided the Prmt1^fl/fl^ mice and Rosa26‐Cre‐ER^T2^ mice. Y.L.J. advised on experiments and provided reagents. C.L. and Y.L.J. wrote the manuscript. J.X.P. and Y.L.J. supervised the entire study. All authors reviewed the manuscript.

## Supporting information



Supporting Information

## Data Availability

The RNA‐sequencing data are deposited at Sequence Read Archive (SRA) repository and are accessible through SRA: PRJNA1163962. The data that support the findings of this study are available from the corresponding author upon reasonable request. Additionally, images in Graphic abstract and Figure 7N were created in BioRender.com (Licence: BioRender.com/v78y802).
