# Supplementary material for: PRMT1 Promotes the Self‐renewal of Leukemia Stem Cells by Regulating Protein Synthesis
Source: Adv Sci (Weinh). 2024 Dec 12;12(5):2308586. doi: 10.1002/advs.202308586 (PMC11791931; doi:10.1002/advs.202308586)
Supplement: Supplementary file 1 — Supporting Information [file ADVS-12-2308586-s001.pdf]

ADVANCED SCIENCE
Open Access

Supporting Information

for *Adv. Sci.*, DOI 10.1002/advs.202308586

PRMT1 Promotes the Self-renewal of Leukemia Stem Cells by Regulating Protein Synthesis

Min Zhou, Yi Huang, Ping Xu, Shuyi Li, Chen Duan, Xiaoying Lin, Shilai Bao, Waiyi Zou*, Jingxuan Pan*, Chang Liu* and Yanli Jin**

Supporting Information

Supplementary methods

Chemicals and Reagents: Type I PRMTs inhibitor MS023 (Cat# HY-19615) and O-propargyl-puromycin (OP-Puro, Cat# HY-15680) were purchased from MedChemExpress (Shanghai, China). 5-Fluorouracil (5-FU, Cat# F6627), tamoxifen (Cat# T5648), puromycin (Cat# P8833), G418 (Cat# G5013), polybrene (Cat# H9268) and EZ-ChIP Kit (Cat# 17-371) were purchased from MilliporeSigma (Shanghai, China). SCF (Cat# 250-03), IL-3 (Cat# 213-13) and IL-6 (Cat# 216-16) were purchased from PeproTech (Rocky Hill, NJ). MethoCult M3434 (Cat# 03434) and MethoCult H4434 (Cat# 04434) were purchased from STEMCELL Technologies (Vancouver, Canada). Human CD34 MicroBead Kit (Cat# 130-046-702), human CD38 MicroBead Kit (Cat# 130-092-263) and mouse CD117 MicroBeads (Cat# 130-091-224) were purchased from Miltenyi Biotec (Auburn, CA). FastKing cDNA synthesis Kit (Cat# KR118-02) and FastFire qPCR PreMix SYBRGreen (Cat# FP207-02) were purchased from TIANGEN (Beijing, China). Trizol (Cat# 10296010CN) was purchased from Thermo Fisher Scientific (Shanghai, China).

Cell Culture: 293T and Plat-E cells were obtained from the American Type Culture Collection (ATCC, Manassas, VA) and Cell Biolabs Inc (San Diego, CA), respectively. Cells were cultured in Dulbecco's modified Eagle’s medium (DMEM) containing 10% FBS at 37°C in a humidified incubator with 5% CO_2. The cell lines were

authenticated by using short tandem repeat matching analysis half a year. No mycoplasma contamination was detected.

Quantitative Real-time PCR (qRT-PCR) Analysis: Total mRNA was extracted from cells using Trizol reagent according to the manufacturer's instruction. Equal amounts of mRNA were used for reverse transcription to synthesize cDNA by using a FastKing cDNA synthesis Kit. qRT-PCR was performed with the FastFire qPCR PreMix SYBRGreen. *GAPDH* or *Gapdh* was used as an endogenous reference. The primer sequences were listed in Table S3.

Western Blotting Analysis: Cells were lysed in RIPA buffer (1× PBS, 1% NP-40, 0.5% sodium deoxycholate, 0.1% SDS) supplemented with protease and phosphatase inhibitors to obtain whole-cell lysates. Protein concentration was detected by using Pierce BCA Protein Assay Kit (Cat# 23227, Thermo Fisher Scientific). Subsequently, equal protein samples were analyzed by sodium dodecyl sulfate polyacrylamide gel electrophoresis (SDS-PAGE) gels, and transferred to nitrocellulose (NC) membranes following blocked in 5% non-fat milk at room temperature for 1 h. The membranes were incubated with the corresponding primary antibodies overnight at 4°C. Protein levels were detected by using the Odyssey infrared imaging system (LI-COR, Lincoln, Nebraska) after incubating with the indicated secondary antibodies for 1 h. The information of antibodies was summarized in Table S4.

Supplementary figures and figure legends

Figure S1

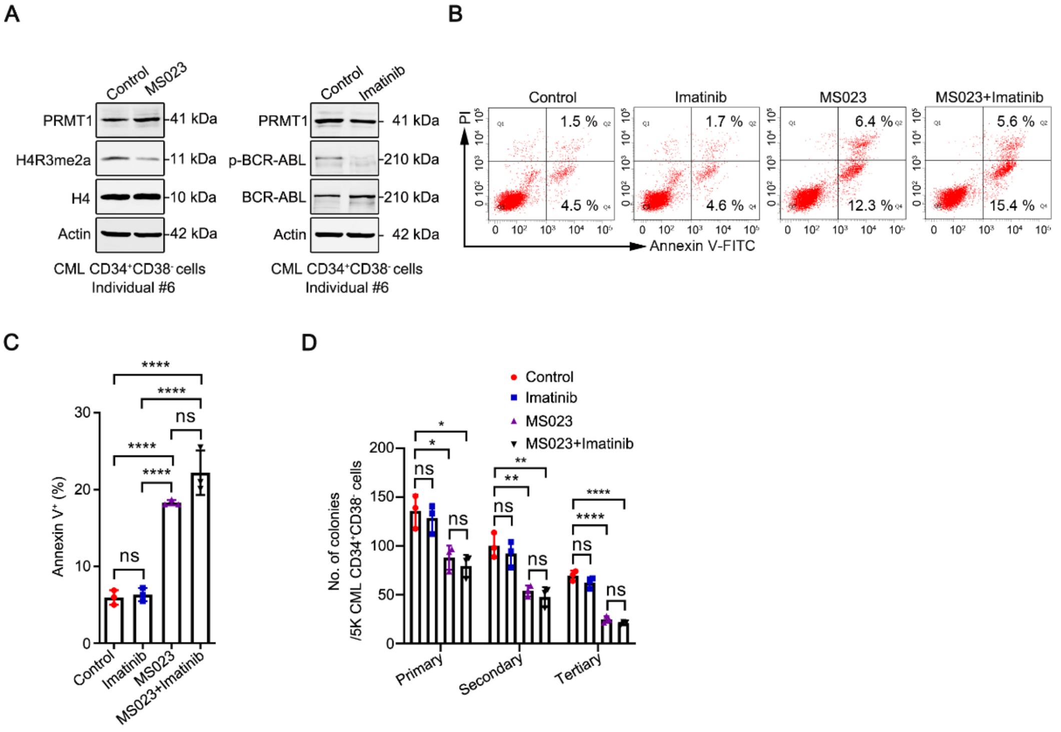

Figure S1. Pharmacological inhibition of PRMT1 induces apoptosis and inhibits the serially plating ability of CML LSCs. (A) Validation of the pharmacological effect of MS023 on methyltransferase activity of PRMT1 or imatinib on tyrosine kinase activity of BCR-ABL in CML LSCs. Primary CML $CD34^{+}CD38^{-}$ cells were treated with MS023 (50 μM) or imatinib (2.5 μM) for 48 h, respectively. The protein levels of PRMT1 and H4R3me2a, as well as phospho-BCR-ABL (p-BCR-ABL) and BCR-ABL were examined by Western blotting analysis. **(B and C)** MS023 treatment induced apoptosis in CML LSCs. Primary CML $CD34^{+}CD38^{-}$ cells (n = 3) were treated with MS023 (50 μM) ± imatinib (2.5 μM) for 48 h, the apoptotic cells (Annexin V^{+}) were measured by flow cytometry after staining with Annexin V-FITC and PI. Representative flow cytometry histograms **(B)** and quantitative results **(C)** for apoptosis were shown. **(D)** MS023 treatment suppressed the serially plating ability of CML LSCs. Primary CML $CD34^{+}CD38^{-}$ cells (n = 3) were treated with MS023 (50 μM) ± imatinib (2.5 μM) for 48 h and subjected to 3 rounds of CFC/replating assay. Data are represented as means ± SEM. *$p < 0.05$, **$p < 0.01$, ****$p < 0.0001$, ns, not significant, by one-way ANOVA with Tukey's test (C and D).

Figure S2

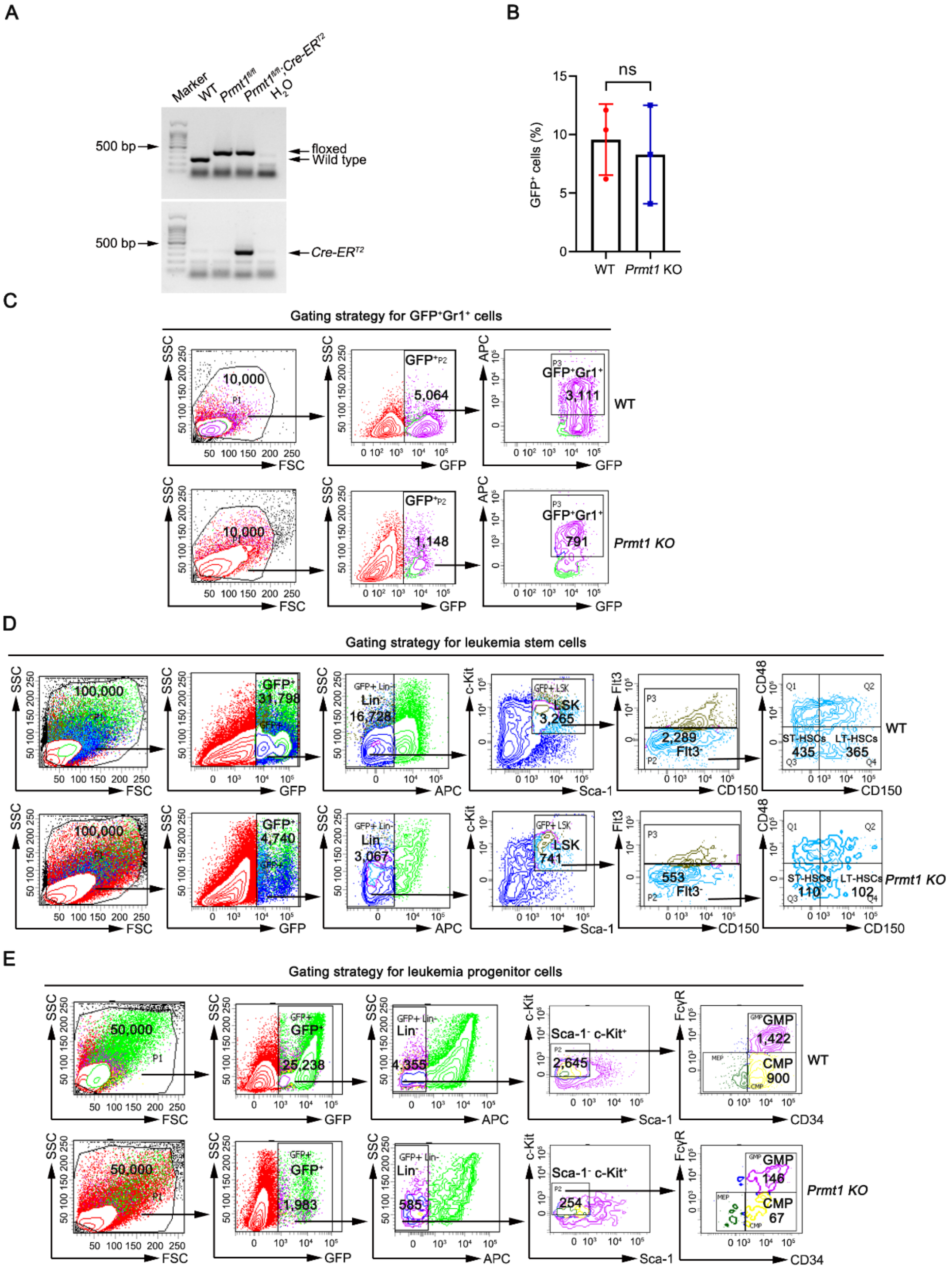

A
Marker
WT
Prmt1fl/fl
Prmt1fl/fl;Cre-ERT2
H2O
500 bp
floxed
Wild type
500 bp
Cre-ERT2
B
ns
GFP+ cells (%)
WT
Prmt1 KO
C
Gating strategy for GFP+Gr1+ cells
SSC
FSC
GFP
APC
10,000
GFP+
5,064
GFP+Gr1+
3,111
WT
10,000
GFP+
1,148
GFP+Gr1+
791
Prmt1 KO
D
Gating strategy for leukemia stem cells
100,000
GFP+
31,798
Lin-
16,728
LSK
3,265
2,289
Flt3-
ST-HSCs
435
LT-HSCs
365
WT
100,000
GFP+
4,740
Lin-
3,067
LSK
741
553
Flt3-
ST-HSCs
110
LT-HSCs
102
Prmt1 KO
c-Kit
Sca-1
Flt3
CD150
CD48
E
Gating strategy for leukemia progenitor cells
50,000
GFP+
25,238
Lin-
4,355
Sca-1- c-Kit+
2,645
GMP
1,422
CMP
900
WT
50,000
GFP+
1,983
Lin-
585
Sca-1- c-Kit+
254
GMP
146
CMP
67
Prmt1 KO
FcγR
CD34

Figure S2. *Prmt1* KO eliminates LSCs in CML mice. (A) Inducible *Prmt1* KO was confirmed by PCR analysis. Genomic DNA was extracted from mouse tails. Representative PCR analysis of WT, *Prmt1* floxed alleles, *Prmt1* floxed alleles and *Cre-ER*^{T2} alleles were shown. **(B)** Flow cytometry analysis of the transduction efficiency of BCR-ABL-iCre-GFP retrovirus in BM cells from WT or *Prmt1* KO mice. *n* = 3 mice per group. **(C-E)** Flow cytometry gating strategies for analysis of populations of leukemia (GFP^{+}) cells and myeloid (GFP^{+}Gr1^{+}) cells **(C)**, leukemia stem cells including GFP^{+}LSK cells (GFP^{+}Lin^{-}Sca-1^{+}c-Kit^{+}), GFP^{+}LT-HSCs (GFP^{+}LSK Flt3^{-}CD150^{+}CD48^{-}) and GFP^{+}ST-HSCs (GFP^{+}LSK Flt3^{-}CD150^{-}CD48^{-}) **(D)**, as well as leukemia progenitor cells including GFP^{+}GMP (GFP^{+}Lin^{-}Sca-1^{-}c-Kit^{+}CD34^{+}FcγRII/III^{high}) and GFP^{+}CMP cells (GFP^{+}Lin^{-}Sca-1^{-}c-Kit^{+}CD34^{+}FcγRII/III^{low}) **(E)** in BM cells from CML mice were shown. Data are represented as means ± SEM. ns, not significant, by Student's t test (B).

Figure S3

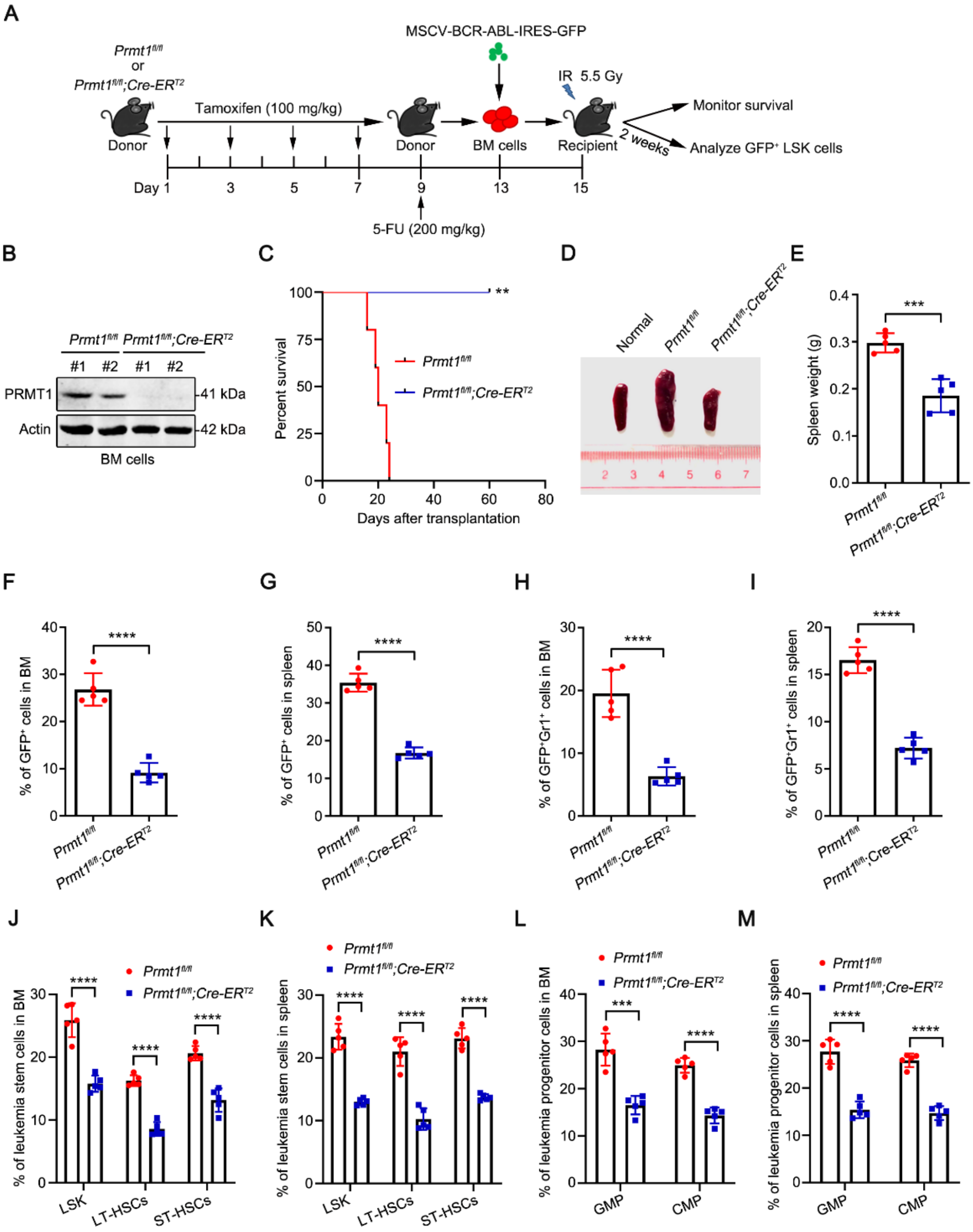

Figure S3. PRMT1 is responsible for the development of BCR-ABL–driven murine CML. (A) Experimental strategy to evaluate the effect of *Prmt1* deletion on leukemogenesis *in vivo*. *Prmt1*$^{fl/fl}$ or *Prmt1*$^{fl/fl}$;*Cre-ER*^{T2} donor mice were treated with tamoxifen (100 mg/kg/gavage, every other day) on day 1, 3, 5, 7 before 5-FU treatment. The BM cells from *Prmt1*$^{fl/fl}$ or *Prmt1*$^{fl/fl}$;*Cre-ER*^{T2} donor mice were transduced with the MSCV-BCR-ABL-IRES-GFP retrovirus and transplanted into sublethally irradiated (550 cGy) recipients to induce CML. **(B)** Western blotting analysis of the protein level of PRMT1 in BM cells from *Prmt1*$^{fl/fl}$ and *Prmt1*$^{fl/fl}$;*Cre-ER*^{T2} mice treated with tamoxifen. **(C)** Kaplan–Meier survival curves of recipients were plotted. n = 5 mice per group. **(D and E)** *Prmt1* deletion suppressed the splenomegaly. The size **(D)** and weight **(E)** of spleens on day 14 post transplantation. n = 5 mice per group **(E)**. The percentages of GFP^{+} cells **(F and G)** and GFP^{+}Gr1^{+} cells **(H and I)** in BM and spleen were analyzed by flow cytometry. n = 5 mice per group. The proportions of GFP^{+}LSK cells, GFP^{+}LT-HSCs, and GFP^{+}ST-HSCs in BM **(J)** and spleen **(K)** were analyzed by flow cytometry. n = 5 mice per group. The populations of GFP^{+}GMP and GFP^{+}CMP cells in BM **(L)** and spleen **(M)** were analyzed by flow cytometry. n = 5 mice per group. Data are represented as means ± SEM. **p < 0.01, ***p < 0.001, ****p < 0.0001, by Student’s t test (E-M) or log-rank test (C).

Figure S4

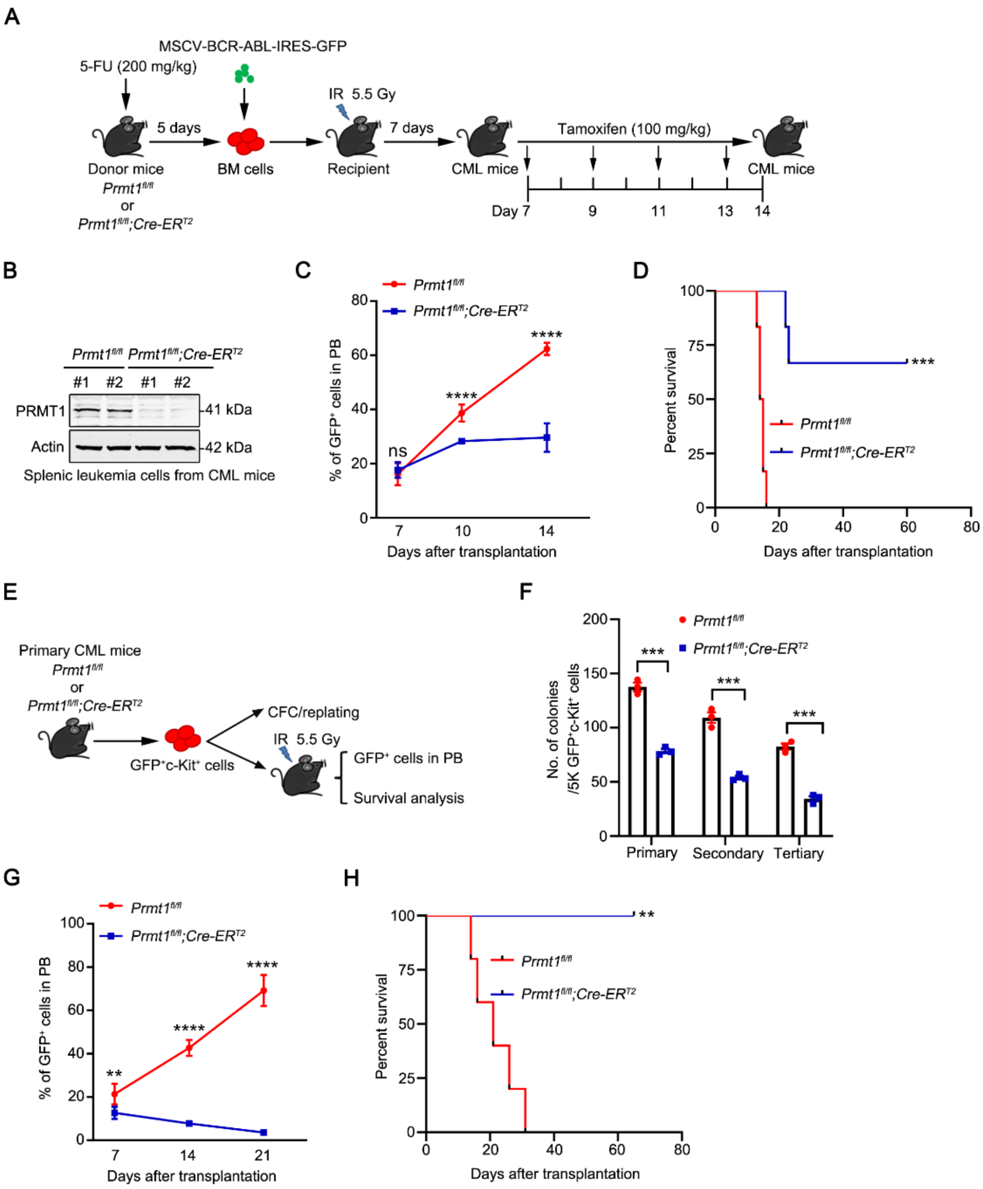
A
MSCV-BCR-ABL-IRES-GFP
5-FU (200 mg/kg)
IR 5.5 Gy
5 days
7 days
Tamoxifen (100 mg/kg)
Donor mice
Prmt1fl/fl
or
Prmt1fl/fl;Cre-ERT2
BM cells
Recipient
CML mice
CML mice
Day 7
9
11
13
14
B
Prmt1fl/fl
Prmt1fl/fl;Cre-ERT2
#1
#2
#1
#2
PRMT1
41 kDa
Actin
42 kDa
Splenic leukemia cells from CML mice
C
Prmt1fl/fl
Prmt1fl/fl;Cre-ERT2
% of GFP+ cells in PB
ns
****
****
Days after transplantation
D
Percent survival
***
Prmt1fl/fl
Prmt1fl/fl;Cre-ERT2
Days after transplantation
E
Primary CML mice
Prmt1fl/fl
or
Prmt1fl/fl;Cre-ERT2
GFP+c-Kit+ cells
CFC/replating
IR 5.5 Gy
GFP+ cells in PB
Survival analysis
F
Prmt1fl/fl
Prmt1fl/fl;Cre-ERT2
No. of colonies
/5K GFP+c-Kit+ cells
***
***
***
Primary
Secondary
Tertiary
G
Prmt1fl/fl
Prmt1fl/fl;Cre-ERT2
% of GFP+ cells in PB
**
****
****
Days after transplantation
H
Percent survival
**
Prmt1fl/fl
Prmt1fl/fl;Cre-ERT2
Days after transplantation

Figure S4. *Prmt1* KO inhibits the propagation of leukemia and impairs the self-renewal of LSCs. (A-C) *Prmt1* KO inhibited the propagation of leukemia. Experimental strategy to evaluate the effect of *Prmt1* deletion on propagation of leukemia. BM cells from 5-FU treated $Prmt1^{fl/fl}$;$Cre\text{-}ER^{T2}$ or $Prmt1^{fl/fl}$ mice were transduced with MSCV-BCR-ABL-IRES-GFP retrovirus and transplanted into sublethally irradiated recipients. The recipients were administered with tamoxifen (100 mg/kg/gavage, every other day) on day 7, 9, 11 and 13 post transplantation **(A)**. Western blotting analysis of the protein level of PRMT1 in splenic leukemia cells from recipients treated with tamoxifen **(B)**. Flow cytometry analysis of the percentage of GFP^+ cells in peripheral blood (PB) from recipient mice on day 7, 10 and 14 post transplantation. $n = 6$ mice per group **(C)**. Kaplan–Meier survival curves of recipients were plotted. $n = 6$ mice per group **(D)**. **(E-H)** *Prmt1* KO impaired the self-renewal of CML LSCs. Experimental strategy to determine the impact of *Prmt1* KO on the self-renewal of LSCs **(E)**. GFP^+c-Kit^+ cells (5,000 cells/well) sorted from primary $Prmt1^{fl/fl}$;$Cre\text{-}ER^{T2}$ or $Prmt1^{fl/fl}$ CML mice were seeded in MethoCult M3434 methylcellulose medium for 3 rounds of CFC/replating assay **(F)**. GFP^+c-Kit^+ cells (2×10^5 cells/mouse) were transplanted into secondary sublethally irradiated (550 cGy) C57BL/6 mice. The percentages of GFP^+ cells in PB on day 7, 14 and 21 post transplantation were detected by flow cytometry **(G)**. Kaplan-Meier survival curves of secondary recipients were shown **(H)**. $n = 5$ mice per group. Data are represented as means ± SEM. $^{**}p < 0.01$, $^{***}p < 0.001$, $^{****}p < 0.0001$, ns, not significant; by Student's t test (C, F and G) or log-rank test (D and H).

Figure S5

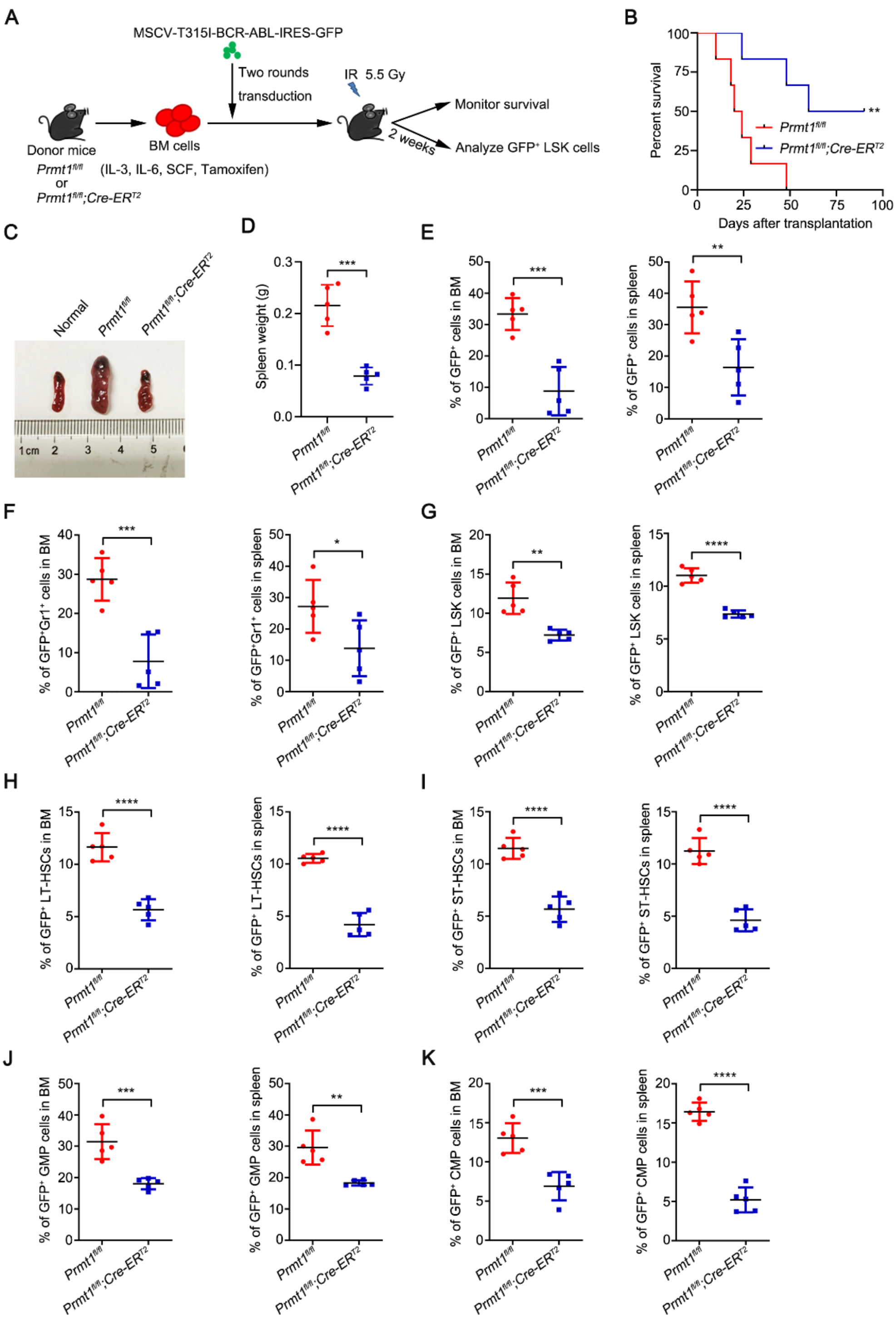
A
MSCV-T315I-BCR-ABL-IRES-GFP
Two rounds transduction
IR 5.5 Gy
Monitor survival
2 weeks
Analyze GFP+ LSK cells
Donor mice
BM cells
Prmt1fl/fl (IL-3, IL-6, SCF, Tamoxifen)
or
Prmt1fl/fl;Cre-ERT2
B
Percent survival
Days after transplantation
Prmt1fl/fl
Prmt1fl/fl;Cre-ERT2
**
C
Normal
Prmt1fl/fl
Prmt1fl/fl;Cre-ERT2
1cm 2 3 4 5
D
Spleen weight (g)
***
E
% of GFP+ cells in BM
% of GFP+ cells in spleen
F
% of GFP+Gr1+ cells in BM
% of GFP+Gr1+ cells in spleen
G
% of GFP+ LSK cells in BM
% of GFP+ LSK cells in spleen
H
% of GFP+ LT-HSCs in BM
% of GFP+ LT-HSCs in spleen
I
% of GFP+ ST-HSCs in BM
% of GFP+ ST-HSCs in spleen
J
% of GFP+ GMP cells in BM
% of GFP+ GMP cells in spleen
K
% of GFP+ CMP cells in BM
% of GFP+ CMP cells in spleen
Prmt1fl/fl
Prmt1fl/fl;Cre-ERT2

Figure S5. Loss of *Prmt1* delays CML development independent of mutational status of BCR-ABL. **(A)** The schema to evaluate the effect of *Prmt1* loss on LSCs in T315I-BCR-ABL-driven CML mouse model. **(B)** Kaplan-Meier survival curves were plotted. n = 6 mice per group. **(C and D)** *Prmt1* deletion effectively relieved splenomegaly. Representative photograph of spleen **(C)** and the weight of spleens were shown. n = 5 mice per group **(D)**. **(E and F)** *Prmt1* deletion decreased leukemia cells in T315I CML mice. The percentages of GFP^+ cells **(E)** and GFP^+Gr1^+ cells **(F)** in BM and spleen were measured by flow cytometry. **(G-I)** *Prmt1* deletion reduced leukemia stem cells in T315I CML mice. The proportions of GFP^+LSK cells **(G)**, GFP^+LT-HSCs **(H)** and GFP^+ST-HSCs **(I)** in BM and spleen were detected by flow cytometry. **(J and K)** *Prmt1* deletion decreased leukemia progenitor cells in T315I CML mice. The populations of GFP^+GMP **(J)** and GFP^+CMP cells **(K)** in BM and spleen were detected by flow cytometry. n = 5 mice per group. *$p < 0.05$, **$p < 0.01$, ***$p < 0.001$, ****$p < 0.0001$, by Student's t test (D-K) or log-rank test (B).

Figure S6

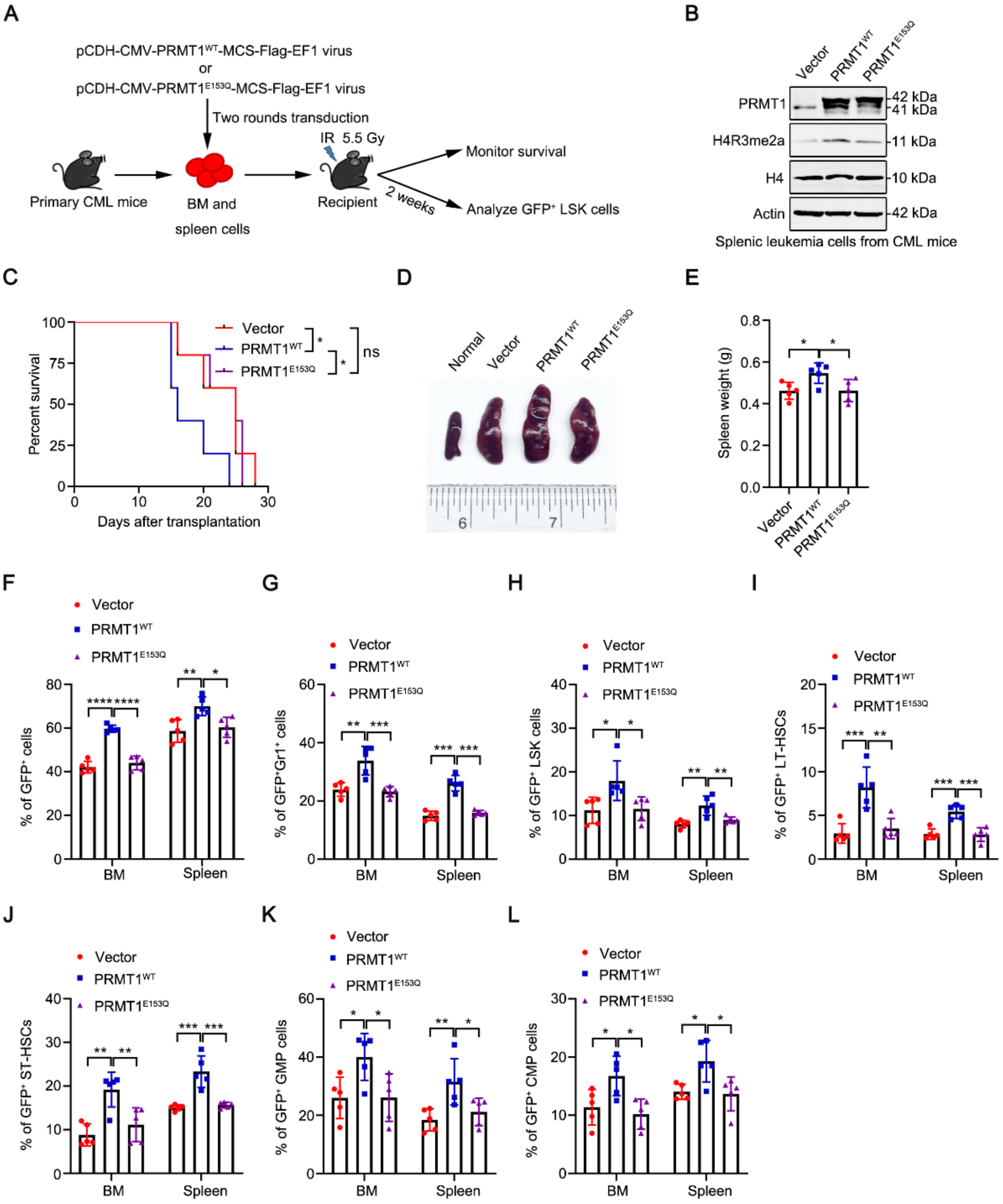
A
pCDH-CMV-PRMT1WT-MCS-Flag-EF1 virus
or
pCDH-CMV-PRMT1E153Q-MCS-Flag-EF1 virus
Two rounds transduction
IR 5.5 Gy
Primary CML mice
BM and spleen cells
Recipient
2 weeks
Monitor survival
Analyze GFP+ LSK cells
B
Vector
PRMT1WT
PRMT1E153Q
PRMT1
H4R3me2a
H4
Actin
42 kDa
41 kDa
11 kDa
10 kDa
42 kDa
Splenic leukemia cells from CML mice
C
Percent survival
Days after transplantation
Vector
PRMT1WT
PRMT1E153Q
ns
D
Normal
Vector
PRMT1WT
PRMT1E153Q
E
Spleen weight (g)
F
% of GFP+ cells
BM
Spleen
G
% of GFP+Gr1+ cells
H
% of GFP+ LSK cells
I
% of GFP+ LT-HSCs
J
% of GFP+ ST-HSCs
K
% of GFP+ GMP cells
L
% of GFP+ CMP cells

Figure S6. PRMT1 regulates LSCs depending on its methyltransferase activity. (A) Experimental strategy to determine whether PRMT1 regulates LSCs in a methyltransferase activity-dependent manner. BM and spleen cells from the primary WT CML mice were transduced with vector, $PRMT1^{WT}$ or $PRMT1^{E153Q}$ lentivirus and transplanted into the secondary recipients to induce CML. **(B)** Western blotting analysis of the protein levels of PRMT1, H4R3me2a and H4 in splenic leukemia cells from secondary recipients. **(C-L)** Overexpression of $PRMT1^{WT}$ rather than $PRMT1^{E153Q}$ aggravated the malignant phenotypes in CML mice. Kaplan-Meier survival curves of secondary recipients were shown. n = 5 mice per group **(C)**. Representative photograph of spleen **(D)** and the weight of spleens **(E)** were shown. n = 5 mice per group. Flow cytometry analysis of the percentages of GFP^+ cells **(F)**, GFP^+Gr1^+ cells **(G)**, GFP^+LSK cells **(H)**, GFP^+LT-HSCs **(I)**, GFP^+ST-HSCs **(J)**, GFP^+GMP **(K)** and GFP^+CMP cells **(L)** in BM and spleen were shown. n = 5 mice per group. Data are represented as means ± SEM. *$p < 0.05$, **$p < 0.01$, ***$p < 0.001$, ****$p < 0.0001$, ns, not significant, by one-way ANOVA with Tukey’s test (E-L) or log-rank test (C).

Figure S7

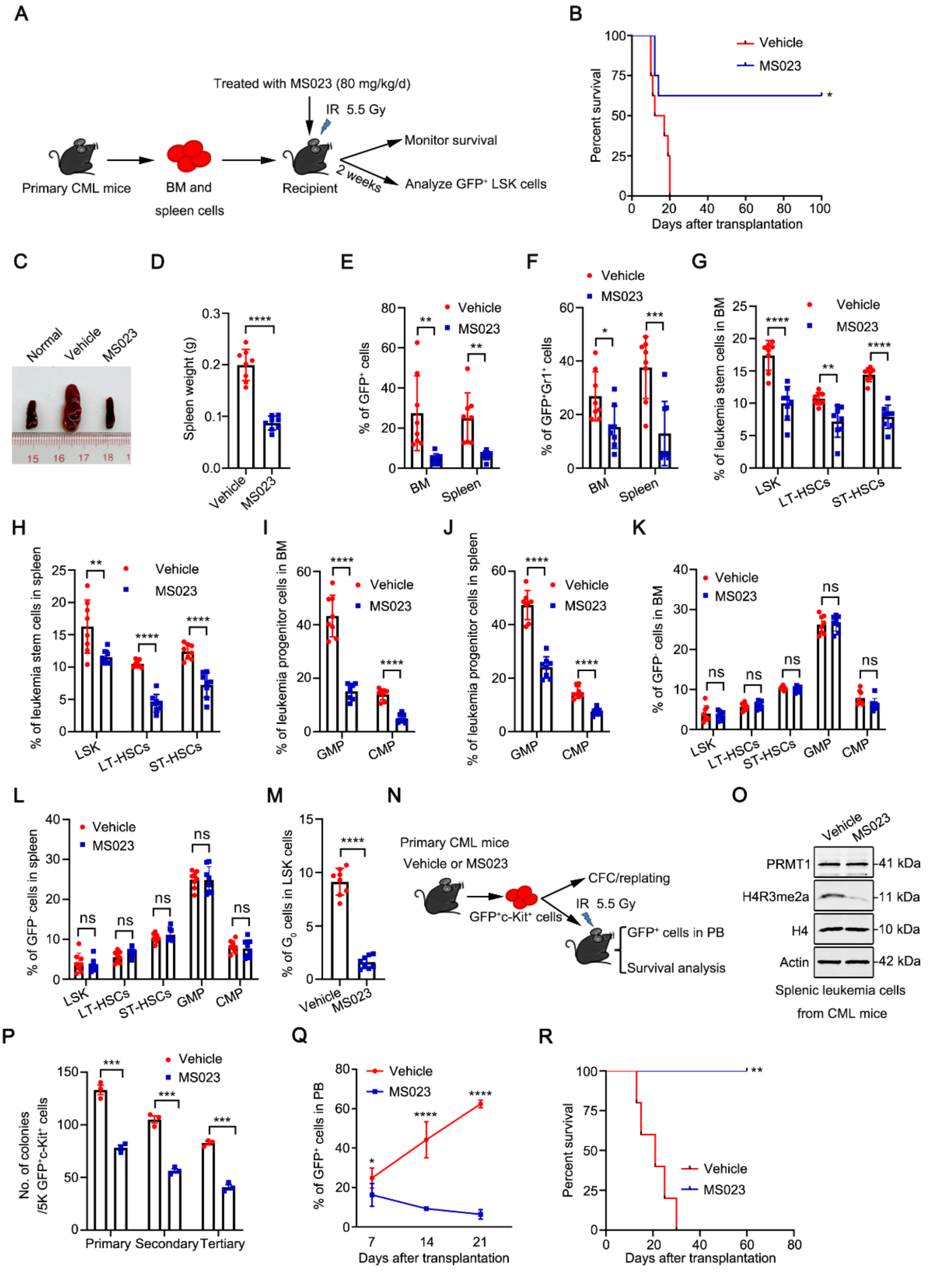

Figure S7. MS023 treatment prolongs the survival of CML mice and impairs the self-renewal of LSCs. (A) Experimental strategy to evaluate the effect of type I PRMTs inhibitor MS023 on LSCs in CML mice. **(B)** Kaplan-Meier survival curves

were plotted. *n* = 8 mice per group. **(C and D)** Representative photograph of spleen **(C)** and the weight of spleens **(D)** were shown. *n* = 8 mice per group. **(E and F)** MS023 treatment decreased leukemia cells in CML mice. The proportions of GFP^+ cells **(E)** and GFP^+Gr1^+ cells **(F)** in BM and spleen were measured by flow cytometry. *n* = 8 mice per group. **(G and H)** MS023 treatment eliminated leukemia stem cells in CML mice. The populations of GFP^+LSK cells, GFP^+LT-HSCs and GFP^+ST-HSCs in BM **(G)** and spleen **(H)** were detected by flow cytometry. *n* = 8 mice per group. **(I and J)** MS023 treatment decreased leukemia progenitor cells in CML mice. The percentages of GFP^+GMP and GFP^+CMP cells in BM (**I**) and spleen (**J**) were detected by flow cytometry. *n* = 8 mice per group. **(K and L)** MS023 treatment had minimal effect on normal hematopoiesis in CML mice. The percentages of GFP^-LSK cells, GFP^-LT-HSCs, GFP^-ST-HSCs, GFP^-GMP, and GFP^-CMP cells in BM (**K**) and spleen (**L**) were detected by flow cytometry. **(M)** MS023 treatment decreased the percentage of quiescent LSK cells. The quiescent LSK cells in BM of CML mice were detected by flow cytometry after staining with Ki67 and Hoechst3342. *n* = 8 mice per group. **(N-R)** MS023 impaired the self-renewal of CML LSCs. Experimental strategy to examine the impact of MS023 on the self-renewal of LSCs **(N)**. Western blotting analysis the protein levels of PRMT1, H4R3me2a and H4 in splenic leukemia cells from vehicle or MS023 treated CML mice **(O)**. GFP^+c-Kit^+ cells (5,000 cells/well) sorted from vehicle or MS023 treated CML mice (*n* = 3) were seeded in MethoCult M3434 methylcellulose medium for CFC/replating assay **(P)**. The GFP^+c-Kit^+ cells (2 $\times 10^5$ cells/mouse) were transplanted into secondary sublethally irradiated (550 cGy) C57BL/6 mice. The percentages of GFP^+ cells in PB of CML mice on day 7, 14, and 21 post transplantation were detected by flow cytometry **(Q)**. Kaplan-Meier survival curves were shown. Vehicle (*n* = 5), MS023 (*n* = 5) **(R)**. Data are represented as means ± SEM. $*p < 0.05$, $**p < 0.01$, $***p < 0.001$, $****p < 0.0001$, ns, not significant, by Student's t test (D-M, P and Q) or log-rank test (B and R).

Figure S8

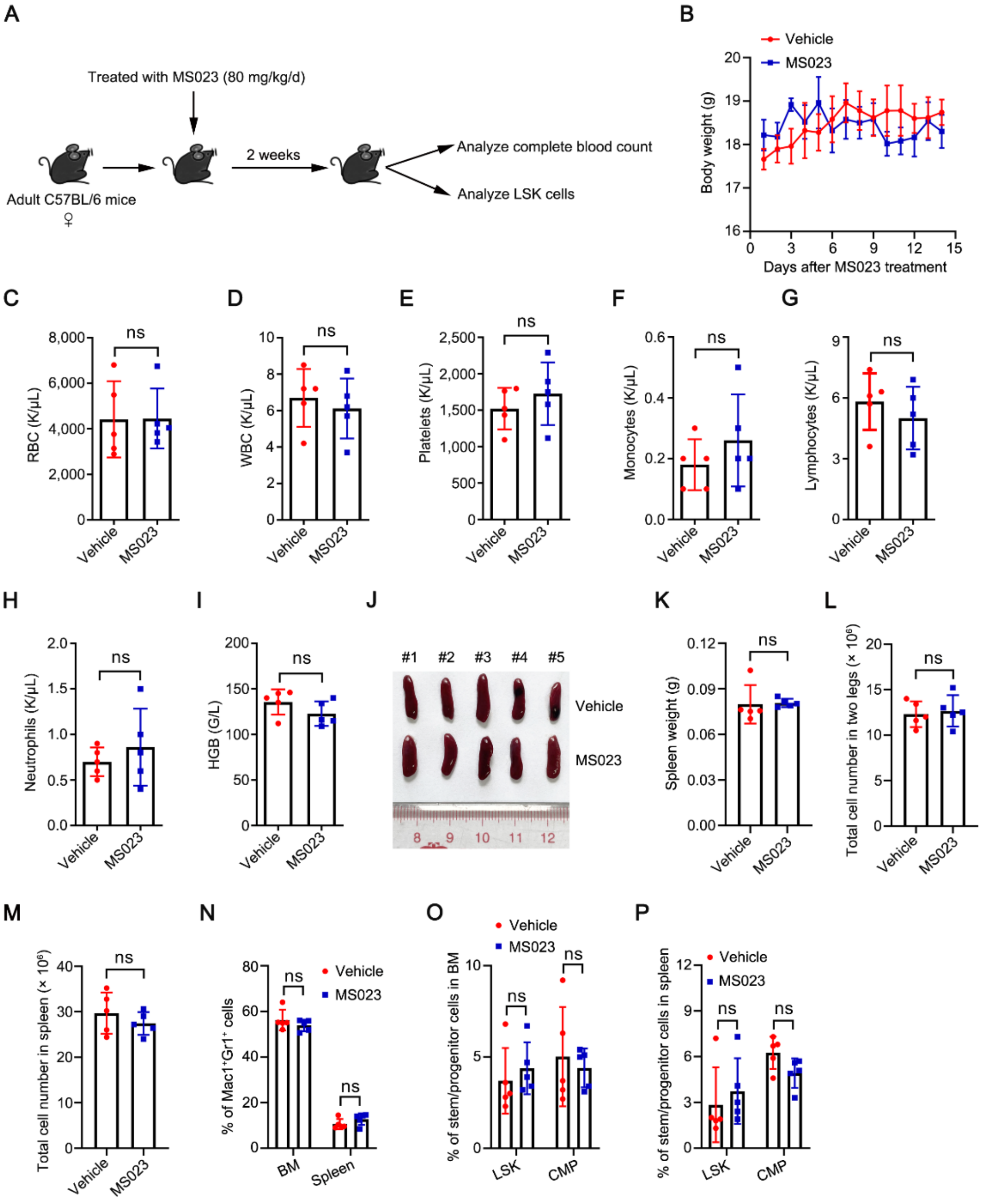
A
Treated with MS023 (80 mg/kg/d)
2 weeks
Adult C57BL/6 mice
♀
Analyze complete blood count
Analyze LSK cells
B
Vehicle
MS023
Body weight (g)
Days after MS023 treatment
C
RBC (K/μL)
D
WBC (K/μL)
E
Platelets (K/μL)
F
Monocytes (K/μL)
G
Lymphocytes (K/μL)
H
Neutrophils (K/μL)
I
HGB (G/L)
J
#1
#2
#3
#4
#5
Vehicle
MS023
K
Spleen weight (g)
L
Total cell number in two legs (× 10⁶)
M
Total cell number in spleen (× 10⁶)
N
% of Mac1⁺Gr1⁺ cells
BM
Spleen
O
% of stem/progenitor cells in BM
LSK
CMP
P
% of stem/progenitor cells in spleen
ns
Vehicle
MS023

Figure S8. Pharmacological inactivation of PRMT1 is minimally detrimental to normal hematopoiesis in adult C57BL/6 mice. (A) Experimental strategy to examine the effect of MS023 on normal hematopoiesis in adult mice. **(B)** The body weight of the mice was monitored. **(C-I)** Complete blood count analysis of PB samples from vehicle and MS023 treated mice. Red blood cells (RBC) **(C)**, white blood cells (WBC) **(D)**, platelets **(E)**, monocytes **(F)**, lymphocytes **(G)**, neutrophils **(H)** and hemoglobin (HGB) **(I)** were analyzed by blood cell counter. *n* = 5 mice per group. **(J and K)** Representative photographs and the weight of spleens were shown. *n* = 5 mice per group. **(L and M)** Total cell number in two legs **(L)** and spleen **(M)** were counted. **(N-P)** The percentages of myeloid ($Mac1^{+}Gr1^{+}$) cells **(N)** and hematopoietic stem/progenitor cells, including LSK cells and CMP cells in BM **(O)** and spleen **(P)** of vehicle and MS023 treated mice were analyzed by flow cytometry. *n* = 5 mice per group. Data are represented as means ± SEM. ns, not significant, by Student's t test (C-I and K-P).

Figure S9

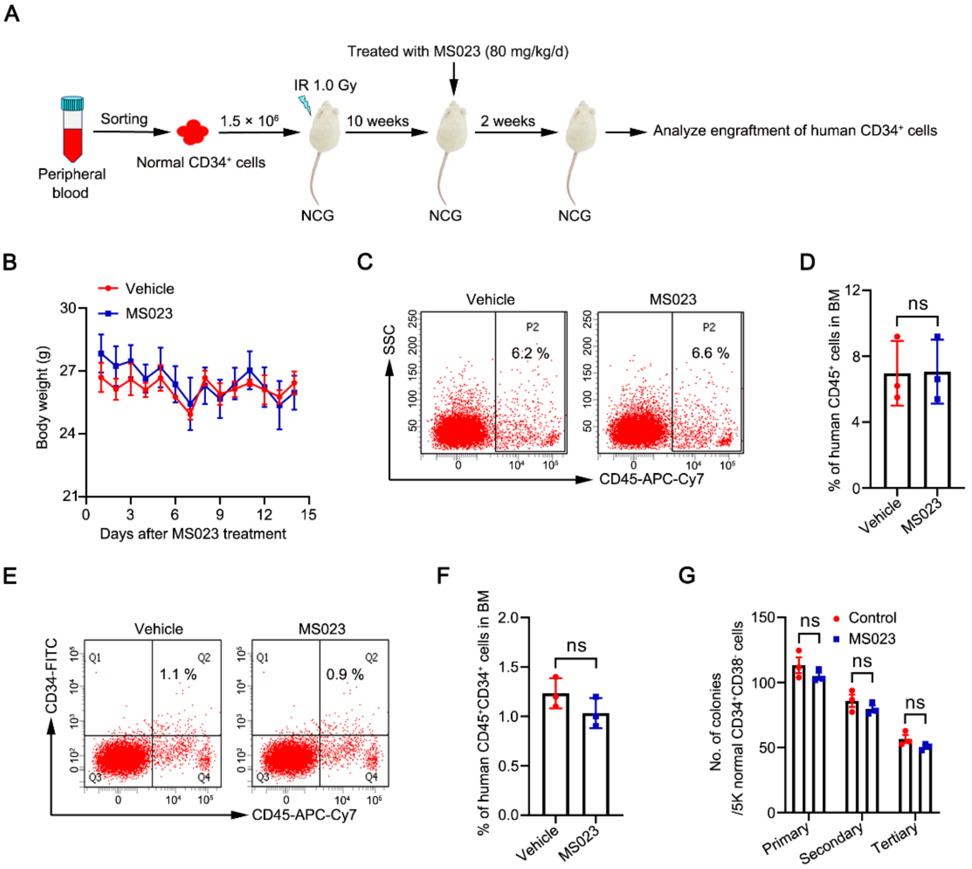
A
Treated with MS023 (80 mg/kg/d)
IR 1.0 Gy
Sorting
1.5 × 10⁶
10 weeks
2 weeks
Analyze engraftment of human CD34⁺ cells
Peripheral blood
Normal CD34⁺ cells
NCG
NCG
NCG
B
Vehicle
MS023
Body weight (g)
Days after MS023 treatment
C
Vehicle
MS023
SSC
P2
6.2 %
6.6 %
CD45-APC-Cy7
D
ns
% of human CD45⁺ cells in BM
Vehicle
MS023
E
Vehicle
MS023
CD34-FITC
Q1
Q2
Q3
Q4
1.1 %
0.9 %
CD45-APC-Cy7
F
ns
% of human CD45⁺CD34⁺ cells in BM
Vehicle
MS023
G
ns
Control
MS023
No. of colonies /5K normal CD34⁺CD38⁻ cells
Primary
Secondary
Tertiary

Figure S9. MS023 has minimal effect on the engraftment of normal CD34$^+$ cells in NOD/ShiLtJGpt-*Prkdc*^{em26Cd52}*Il2rg*^{em26Cd22}/Gpt (NCG) mice. **(A)** Experimental procedure to determine the effect of MS023 on engraftment of normal CD34$^+$ cells in NCG mice. **(B)** The body weight of the mice was monitored. **(C and D)** Representative flow cytometry histograms and quantitative results for human CD45$^+$ cells in BM were shown. *n* = 3 mice per group. **(E and F)** Representative flow cytometry histograms and quantitative results for human CD45$^+$CD34$^+$ cells in BM were shown. *n* = 3 mice per group. **(G)** Primary normal CD34$^+$CD38$^-$ cells (*n* = 3) were treated with MS023 (50 μM) for 48 h and subjected to 3 rounds of CFC/replating assay. Data are represented as means ± SEM. ns, not significant, by Student's t test (D, F and G)

Figure S10

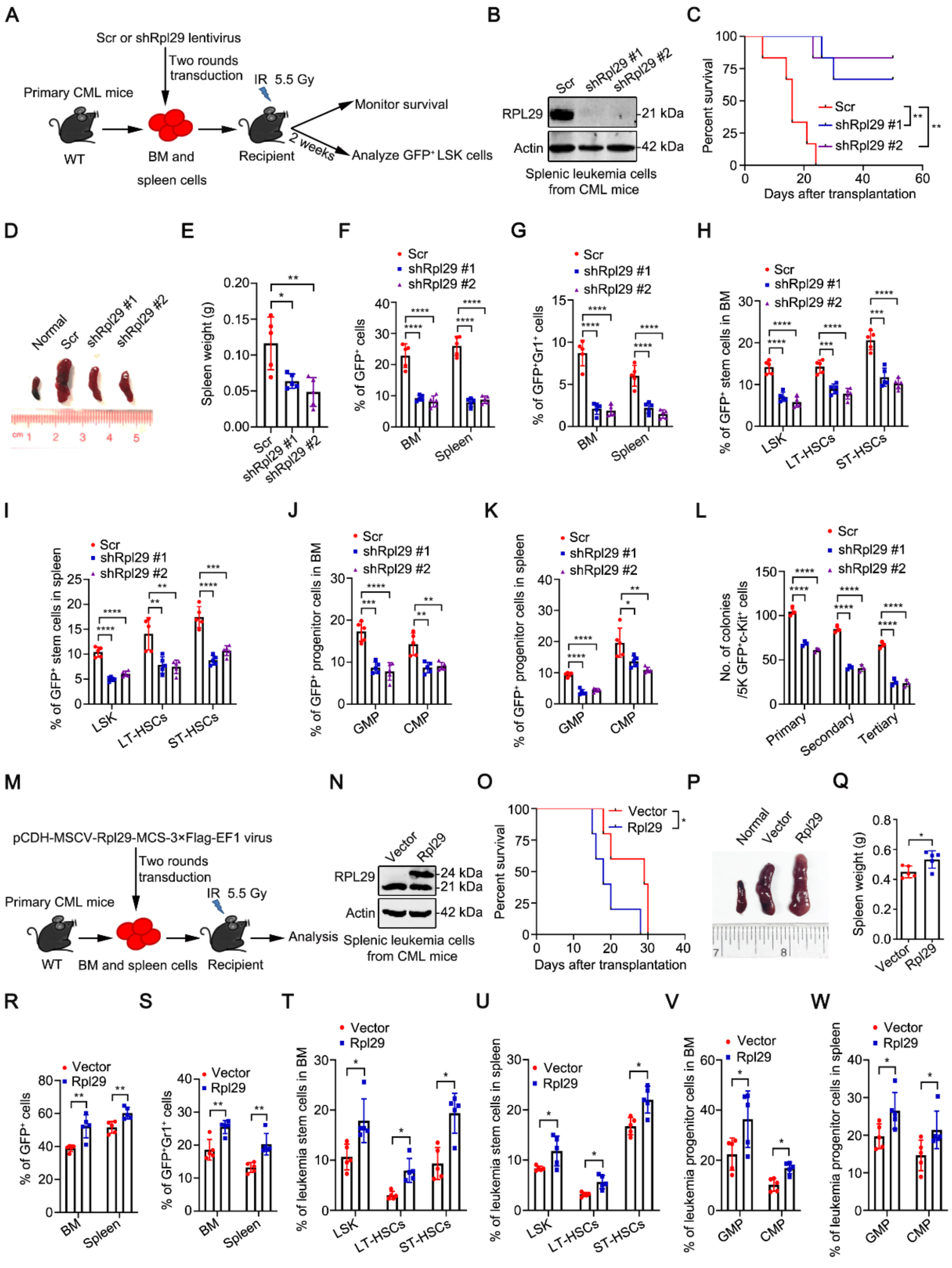
A
Scr or shRpl29 lentivirus
Two rounds transduction
IR 5.5 Gy
Primary CML mice
Monitor survival
2 weeks
Analyze GFP⁺LSK cells
WT
BM and spleen cells
Recipient
B
Scr
shRpl29 #1
shRpl29 #2
RPL29
21 kDa
Actin
42 kDa
Splenic leukemia cells from CML mice
C
Percent survival
Days after transplantation
Scr
shRpl29 #1
shRpl29 #2
D
Normal
Scr
shRpl29 #1
shRpl29 #2
E
Spleen weight (g)
F
% of GFP⁺ cells
BM
Spleen
G
% of GFP⁺Gr1⁺ cells
H
% of GFP⁺ stem cells in BM
LSK
LT-HSCs
ST-HSCs
I
% of GFP⁺ stem cells in spleen
J
% of GFP⁺ progenitor cells in BM
GMP
CMP
K
% of GFP⁺ progenitor cells in spleen
L
No. of colonies /5K GFP⁺c-Kit⁺ cells
Primary
Secondary
Tertiary
M
pCDH-MSCV-Rpl29-MCS-3×Flag-EF1 virus
Two rounds transduction
IR 5.5 Gy
Primary CML mice
Analysis
WT
BM and spleen cells
Recipient
N
Vector
Rpl29
RPL29
24 kDa
21 kDa
Actin
42 kDa
Splenic leukemia cells from CML mice
O
Vector
Rpl29
Percent survival
Days after transplantation
P
Normal
Vector
Rpl29
Q
Spleen weight (g)
R
% of GFP⁺ cells
S
% of GFP⁺Gr1⁺ cells
T
% of leukemia stem cells in BM
U
% of leukemia stem cells in spleen
V
% of leukemia progenitor cells in BM
W
% of leukemia progenitor cells in spleen

Figure S10. RPL29 regulates LSCs and leukemia development in CML mice. (A) Experimental strategy to examine the effect of *Rpl29* knockdown on LSCs in CML mice. BM and spleen cells from the primary CML mice transduced with Scr, sh*Rpl29* #1, or sh*Rpl29* #2 lentivirus were transplanted into irradiated (550 cGy) secondary recipient mice to induce CML. **(B)** RPL29 knockdown in splenic leukemia cells from CML mice was confirmed by Western blotting analysis. **(C)** Kaplan-Meier survival curves. n = 6 mice per group. **(D and E)** Representative photograph of the spleen **(D)** and the weight of spleens **(E)** were shown. n = 5 mice per group. **(F-K)** *Rpl29* knockdown eliminated LSCs in CML mice. The proportions of GFP$^+$ cells (**F**), GFP$^+$Gr1$^+$ cells (**G**), GFP$^+$LSK cells, GFP$^+$ LT-HSCs and GFP$^+$ ST-HSCs (**H and I**), as well as GFP$^+$GMP and GFP$^+$CMP cells **(J and K)** in BM and spleen were measured by flow cytometry. n = 5 mice per group. **(L)** *Rpl29* knockdown suppressed the serially plating capacity of GFP$^+$c-Kit$^+$ cells. GFP$^+$c-Kit$^+$ cells (5,000 cells/well) sorted from Scr, sh*Rpl29* #1, or sh*Rpl29* #2 CML mice (n = 3) were seeded in MethoCult M3434 methylcellulose medium for 3 rounds of CFC/replating assay. **(M)** Experimental strategy to examine the effect of *Rpl29* overexpression on LSCs in CML mice. **(N)** The protein level of RPL29 in splenic leukemia cells from recipients was detected by Western blotting analysis. **(O)** Kaplan-Meier survival curves. n = 5 mice per group. **(P and Q)** Representative photograph of the spleen and the weight of spleens were shown. n = 5 mice per group. **(R-W)** Overexpression of *Rpl29* increases LSCs in CML mice. The proportions of GFP$^+$ cells **(R)**, GFP$^+$Gr1$^+$ cells **(S)**, GFP$^+$LSK cells, GFP$^+$ LT-HSCs and GFP$^+$ ST-HSCs **(T and U)**, as well as GFP$^+$GMP and GFP$^+$CMP cells **(V and W)** in BM and spleen were measured by flow cytometry. n = 5 mice per group. Data are represented as means ± SEM. *$p < 0.05$, **$p < 0.01$, ***$p < 0.001$, ****$p < 0.0001$, by one-way ANOVA with Tukey's test (E-L), Student's test (Q-W) or log-rank test (C and O).

Figure S11

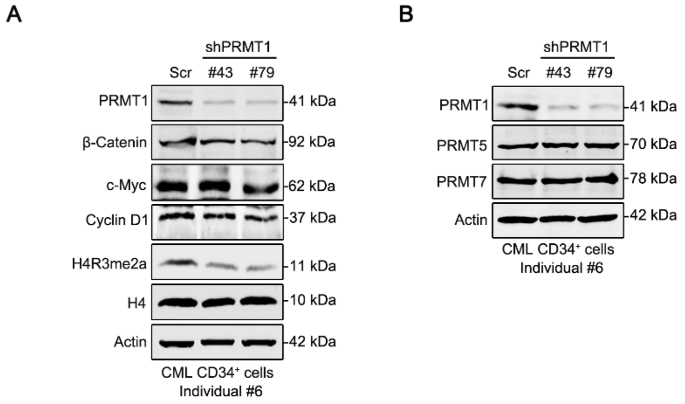

Figure S11. PRMT1 knockdown does not inhibit Wnt/β-catenin signaling and alter the protein levels of PRMT5 or PRMT7 in CML CD34$^+$ cells. Western blotting analysis of the protein levels of β-catenin, c-Myc, and Cyclin D1 **(A)**, as well as PRMT5 and PRMT7 **(B)** in CML CD34$^+$ cells with *PRMT1* knockdown.

Table S1. ***In vivo*** **limiting dilution assay of leukemia stem cells (LSCs) in WT and** ***Prmt1*** **KO CML mice. Related to Figure 2.**

Cell number	Engrafted/tested mice	
	**WT**	***Prmt1*** **KO**
2×10^6	7/7	4/6
1×10^6	7/7	3/7
5×10^5	5/6	2/7
LSCs frequency	1/550,837	1/3,402,081

Table S2. Characteristics of individuals with chronic myeloid leukemia (CML).

Individual Number	Sex/ Age (yr)	Disease stage	Sample	Date of diagnosis	Prior therapy	WBC count (10^9/L)	Blastocytes (%)	BCR-ABL Positive
1	M/28	CP	PB	8/6/2022	Initial	443.72	1%	+
2	F/58	CP	PB	23/2/2022	Initial	434.81	1%	+
3	M/19	CP	PB	7/3/2023	Initial	685.64	ND	+
4	F/23	CP	PB	9/6/2022	Initial	545.42	3%	+
5	M/50	CP	PB	3/11/2022	Initial	178.78	5%	+
6	M/59	CP	PB	12/1/2024	Initial	598.13	1%	+
7	M/33	CP	PB	22/1/2024	Initial	336.55	1%	+
8	M/39	CP	PB	2/9/2023	Initial	192.05	0%	+
9	F/36	CP	PB	31/12/2020	Initial	522.56	4%	+
10	M/40	CP	PB	9/12/2020	Initial	285.60	0%	+
11	M/31	CP	PB	14/7/2022	Initial	250.04	2%	+
12	F/43	CP	PB	8/7/2022	Initial	304.85	1%	+
13	F/36	CP	PB	21/4/2022	Initial	455.61	0%	+
14	M/31	CP	PB	23/8/2022	Initial	626.97	0.5%	+
15	M/30	CP	PB	4/12/2017	Imatinib	371.88	0%	+
16	M/29	CP	PB	30/12/2020	Initial	341.60	1%	+
17	M/22	CP	PB	20/9/2020	Initial	571.05	5%	+
18	F/19	CP	PB	13/11/2020	Initial	321.12	2%	+
19	F/47	CP	PB	13/11/2020	Initial	697.67	1%	+
20	F/71	CP	PB	23/10/2020	Initial	231.38	1%	+

CP: chronic phase; PB: peripheral blood; WBC: white blood cells; ND: not detected.

Table S3. Primers for qRT-PCR analysis.

Gene	Forward primer (5'-3')	Reverse primer (5'-3')
GAPDH	CTCCTCCTGTTCGACAGTCAGC	CCATGGAATTTGCCATGGGTGG
Gapdh	CCCACTAACATCAAATGGGG	CCTTCCACAATGCCAAAGTT
PRMT1	TACACGCACTGGAAGCAGA	GGTTGTTCTTGGCGTTGG
RPL29	CAGCTCAGGCTCCCAAAC	GCACCAGTCCTTCTGTCCTC
Rpl29	GATGCAGGCCAACAATGCAA	CTTAGGCTTCGGTTGGCAGA
Adgrg1	CTGCGGCAGATGGTCTACTTC	CCACACAAAGATGTGAGGCTC
Cd69	GAACAAGACAGCTCCAGCTAC	TATACTGGTGCCATGGTCCTTC
Atf3	AGGCAGGAGCATCCTTTGTC	CTGCTTTGCATAGGACCCCA
Ddit4	GCCGGAGGAAGACTCCTCATA	CATCAGGTTGGCACACAGGT
F2r	TCCGACCGAGCTACTCAGAA	AAGAATGAGCGGGGGTTCAC
Nlrp6	CCAGCTGAGCCAGAATGAAC	GGGAGCAGAGGTGATCCTTT

Table S4. Information of antibodies for Western blotting analysis.

Antibody	Company	Cat #
PRMT1 (1:1000)	Cell Signaling Technology	2449S
H4R3me2a (1:500)	Thermo Fisher Scientific	PA5-96124
H4 (1:1000)	MilliporeSigma	05-858
RPL29 (1:1000)	Abcam	Ab67196
Phospho-c-ABL (Y245) (1:500)	Cell Signaling Technology	2861S
c-ABL (1:500)	Cell Signaling Technology	2862S
PRMT5 (1:1000)	MilliporeSigma	07-405
PRMT7 (1:1000)	Cell Signaling Technology	14762S
β-catenin (1:500)	BD Biosciences	610153
Cyclin D1 (1:500)	Santa Cruz Biotechnology	sc-753
c-Myc (1:500)	BD Biosciences	551101
Flag (1:1000)	Beyotime	AF2852
β-Actin (1:8000)	MilliporeSigma	A5441
goat-anti-mouse 800CW (1:10000)	LI-COR	926-32210
goat-anti-rabbit 800CW (1:10000)	LI-COR	926-32211
